# Collecting Eggs, Not Killing Chickens: Why Stem Cell Secretome and Exosomes Are Redefining Regenerative Medicine for Healthspan Extension

**DOI:** 10.3390/biomedicines14040854

**Published:** 2026-04-09

**Authors:** John A. Dangerfield, Christoph Metzner

**Affiliations:** 1 Miskawaan Health Group, 2415/4 New Phetchaburi Road, Bangkapi, Bangkok 10310, Thailand; 2 Institute of Biotechnology, IMC Krems University of Applied Sciences, Kasernstrase 60, 3500 Krems, Austria; christoph.metzner@imc.ac.at

**Keywords:** regenerative medicine, mesenchymal stem cells, secretome, exosomes, neonatal stem cells, longevity, functional medicine, precision medicine

## Abstract

Regenerative medicine is becoming more widely integrated with longevity-oriented and preventive care as populations age and chronic degenerative diseases burden healthcare systems. Mesenchymal stem cell (MSC) therapies have progressed from experimental interventions to approved products, yet scalability, safety, cost, and regulatory complexity constrain widespread implementation in medical wellness contexts. The predominant therapeutic effects of MSCs are mediated via paracrine mechanisms, leading to cell-free approaches based on the MSC secretome—a complex mixture of bioactive factors including all types of biomolecules and assemblies thereof, such as exosomes. These acellular products offer compelling advantages: multiple batches from single-donor sources, standardized dosing, reduced allogeneic cell risks, and shorter outpatient-compatible administration. Preclinical and clinical data indicate that secretome-based products exert potent regenerative effects in osteoarthritis, chronic wounds, stroke, traumatic brain injury, and neurodegenerative diseases. This review examines the evolution from cell-based to cell-free regenerative strategies, focusing on human umbilical cord Wharton’s jelly MSC secretome for precision longevity medicine. It compares MSC therapies with secretome- and exosome-based formulations across mechanistic, manufacturing, safety, practical and regulatory dimensions. Regional perspectives highlight Southeast Asia, and especially Thailand, as an emerging regenerative-longevity hub. Finally, it outlines the preventive patient journey integrating cell-free interventions within multi-modal programs aimed at extending healthspan.

## 1. Regenerative Medicine as a Longevity Tool: Challenges and Opportunities

Rapid demographic aging and the global rise in chronic, non-communicable diseases have inspired intense interest in strategies that can maintain function and quality of life rather than simply extending the lifespan [1,2]. This has fueled the growth of overlapping domains in medicine variously labeled “longevity,” “anti-aging,” “preventive,” “functional” and “precision,” which frequently converge on a shared ambition [3,4,5,6]. Within this landscape, regenerative medicine occupies a pivotal position by offering tools that not only alleviate symptoms but also seek to restore or preserve tissue integrity, function and physiological resilience.

Over the past two decades, mesenchymal stem cell (MSC)-based therapies have moved from experimental concepts to clinical reality, with several products approved in major jurisdictions for indications such as graft-versus-host disease, complex perianal fistulae in Crohn’s disease, and musculoskeletal degenerative conditions [7,8,9].

Despite these advances, conventional MSC therapies face persistent challenges that limit their utility for widespread, preventive or wellness-oriented use. Manufacturing live cell products at scale remains technically demanding and costly, particularly when stringent quality, potency and safety criteria are applied [8,10,11]. High-dose systemic infusions of allogeneic cells raise theoretical and practical safety concerns, including microvascular obstruction, ectopic tissue formation and immunological responses, necessitating prolonged administration times and close medical supervision [12,13,14,15]. In parallel, regulatory agencies continue to grapple with how best to classify, evaluate and monitor cell-based interventions, leading to heterogeneous pathways and, in some regions, restricted access outside formal clinical trials [15,16].

At the same time, a substantial body of preclinical and translational research has converged on the observation that MSCs exert much of their therapeutic effect through the secretion of bioactive factors rather than durable engraftment or transdifferentiation. Conditioned media from MSC cultures, often referred to as the MSC secretome, contains a complex mixture of soluble proteins, lipids, nucleic acids and extracellular vesicles (EVs), including exosomes and other vesicle subtypes [17], which together orchestrate immunomodulatory, pro-angiogenic, anti-apoptotic and pro-regenerative effects in target tissues [18,19,20,21,22,23]. Studies using human Wharton’s jelly MSC-derived (WJMSC) secretome and conditioned media have demonstrated greatly accelerated wound healing, enhanced re-epithelialization and modulation of fibrotic responses in both in vitro and in vivo models, underscoring the regenerative potential of acellular products derived from perinatal tissues [24,25,26,27].

The recognition of these paracrine mechanisms has given rise to a new conceptual paradigm often summarized as “stem cell therapy without the cells,” in which the focus shifts from delivering viable cells to harnessing and standardizing their secreted products [21]. Secretome- and exosome-based therapeutics offer several theoretical advantages in the context of longevity-oriented and preventive medicine: they can be manufactured from well-characterized secretome-producing cell lines, aliquoted and stored for off-the-shelf use [28,29,30]; dosing can be more precisely controlled; and the absence of viable cells reduces certain safety and regulatory concerns, particularly for repeat administration in individuals who may be relatively well but at elevated risk of age-related decline [12,13].

Concurrently, medical wellness and health-tourism hubs, including those in Southeast Asia (and particularly Thailand), have become focal points for the development and delivery of longevity-oriented programs that integrate advanced diagnostics, lifestyle interventions and, in some settings, regenerative therapies [16,31,32]. These ecosystems provide a practical testbed for implementing preventive, multi-modal, regenerative strategies in an international clientele, although they also highlight tensions between innovation, evidence, regulation and commercialization. Understanding how cell-free regenerative products such as MSC secretome and exosomes can be responsibly deployed within such frameworks is becoming increasingly important for clinicians, regulators and patients alike [6,19,23,33].

Against this backdrop, the present narrative review aims to synthesize current knowledge on MSC-based and cell-free regenerative approaches, with a specific focus on secretome and exosomes in the context of preventive and precision medicine for longevity and chronic disease relevant to aging [4]. The manuscript first delineates the conceptual and terminological landscape linking regenerative medicine with longevity, preventive and precision care, before examining the biological foundations of MSC secretome and exosomes and comparing them with traditional cell therapies with regard to their mechanistic, manufacturing and safety properties, as practical and regulatory dimensions. It then explores emerging evidence for applications in key age-associated conditions, including central metabolic and nervous system disorders, and considers regional regulatory and practice perspectives, paying particular attention to Southeast Asia and Thailand as an example of an evolving regenerative-longevity hub [31,32]. It briefly touches on the evolving impact of preventative approaches on medical insurance strategies. Finally, it outlines a model preventive “patient journey” in which regenerative cell-free interventions are integrated into comprehensive, multi-modal programs aimed at extending the healthspan and enhancing resilience throughout later life.

Beyond summarizing mechanisms, this review synthesizes data contrasting cell-free and cell-based regenerative strategies, integrating three dimensions that are usually treated separately: (i) the rationale for prioritizing MSC-derived secretome and exosomes over live MSC therapies, building on prior work that highlights the safety and practical advantages of cell-free approaches; (ii) bioprocess and manufacturing considerations for scalable, GMP-compatible production of MSC secretome and EV-based products, extending recent reviews that focus on standardization and yield optimization; and (iii) the practical integration of these cell-free modalities into preventive and longevity-oriented care pathways, a dimension that has been only briefly touched on in existing exosome and secretome reviews [34].

## 2. Conceptual Framework: From Longevity to Regenerative Precision Medicine

The terminology around slowing aging and preventing chronic disease has expanded to include overlapping concepts such as longevity, anti-aging, preventive, functional, and precision medicine, creating blurred conceptual and regulatory boundaries despite their shared focus on proactive, individualized care. For this review, longevity medicine can be treated as an advanced form of personalized preventive medicine that uses cutting-edge aging biomarkers to extend the healthspan, particularly in the context of regenerative, cell-free therapies [3,4,6,35].

In practice, this often involves the integration of multi-omic profiling, detailed phenotyping and longitudinal monitoring to guide interventions that modulate biological aging processes such as inflammation, senescence, metabolic dysfunction and neurodegeneration [6,36]. “Functional medicine,” by contrast, arises from a systems-biology perspective that seeks to identify and address the root causes of chronic illness across domains such as nutrition, gut health, environmental exposures and psychosocial stress, typically through a combination of lifestyle, nutraceutical and sometimes pharmacological interventions [37]. “Anti-aging medicine” is a broader and more variably used term, encompassing evidence-based interventions as well as more speculative practices whose proposed mechanisms and claimed benefits, despite their popularity, remain weakly supported or unproven, and cosmetically oriented practices; as such, it is less precise and is sometimes viewed critically in academic contexts.

“Precision medicine” and the related concept of “precision health” emphasize tailoring interventions to specific biological, environmental and lifestyle characteristics, originally with a strong focus on genomics but now extending to multi-dimensional data streams and digital phenotyping [35,37,38]. In older adults, precision medicine approaches are now seen as a means to improve care by integrating detailed risk stratification, biomarker-guided therapy and continuous monitoring into routine clinical decision-making [36]. When applied to the domain of aging, the convergence of longevity medicine and precision medicine yields what some have termed “precision longevity,” an iterative model of care based on early detection, prevention and deep personalization of interventions aimed at preserving function and delaying or preventing the onset of age-related disease [4,36,37,38].

Within this conceptual ecosystem, regenerative medicine can be framed as a set of enabling technologies and therapeutic strategies that directly address structural and functional decline at the tissue and organ level. These include cell-based therapies, cell-free products such as secretome and exosomes, tissue engineering, biomaterials and emerging modalities such as in vivo reprogramming [8,15,26]. In the context of longevity-oriented precision care, regenerative interventions play two complementary roles. First, they can be deployed reactively to repair established damage; for example, in patients with osteoarthritis, non-healing wounds or post-stroke neurorehabilitation [4,24,39,40,41,42]. Second, they can be used proactively as part of ongoing care to strengthen tissue, regulate inflammation and immune function, and potentially slow the buildup of age-related problems in people at higher risk [4,36,43].

To avoid terminological proliferation, this review adopts “longevity-oriented precision regenerative medicine” (LPRM) as its core conceptual frame. This term underscores four key elements. First, healthspan is the primary outcome of interest, integrating symptomatic relief with preservation of function and independence [4,37]. Second, the precision element reflects the use of individualized risk profiling, biomarker panels and functional assessments to guide treatment selection, dosing and sequencing over time [35,36,37]. Third, the regenerative component highlights therapies that directly engage repair and remodeling pathways at the cellular and tissue levels [8,18]. Finally, the term implicitly allows for the inclusion of both cell-based and cell-free products, while the focus of this review is on the latter.

From a practical standpoint, LPRM can be envisaged as a patient journey comprising several stages culminating in a personalized plan (detailed in Section 7).

Secretome- and exosome-based products fit naturally within this framework. Their safety profile, potential for standardized manufacturing and suitability for outpatient administration make them particularly attractive for iterative use in individuals who may not meet criteria for severe disease but who exhibit early signs of decline or elevated risk based on biomarker and functional assessments [15,21,28]. In addition, their pleiotropic mechanisms—spanning modulation of inflammation, angiogenesis, extracellular matrix remodeling and neurotrophic support—align well with the multi-system pathophysiology of aging, where single-target interventions often fail to produce meaningful functional gains [18,19,21,33]. Accordingly, positioning secretome or purified EVs as central tools within LPRM provides a coherent conceptual scaffold for the subsequent, more detailed analysis of their comparative advantages over traditional cell therapies in the management of age-related chronic conditions.

## 3. Biological Foundations: MSCs, Secretome and Exosomes

**Figure 1:**
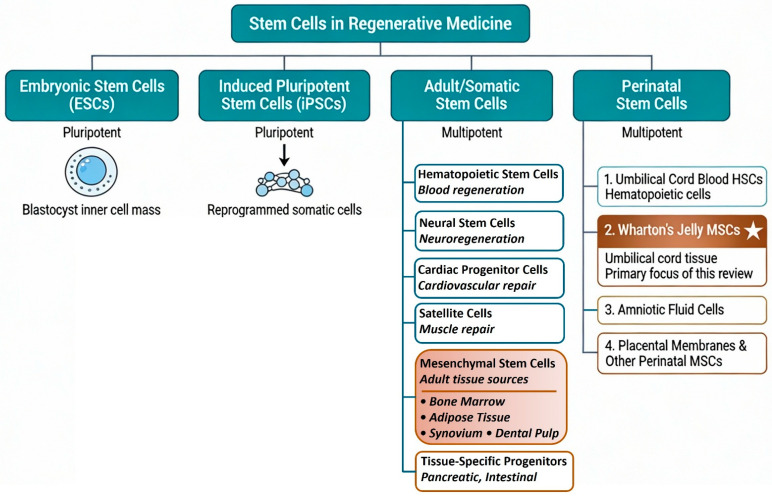
Classification of stem cell types in regenerative medicine. Stem cells used in regenerative therapies can be broadly categorized based on their developmental origin and differentiation potential. Embryonic stem cells (ESCs) and induced pluripotent stem cells (iPSCs) are pluripotent, which means that they are capable of differentiating into all three germ layers. Adult/somatic stem cells include hematopoietic stem cells (HSCs) for blood and immune system regeneration, neural stem cells (NSCs) for neurological applications, cardiac stem/progenitor cells for cardiovascular repair, satellite cells for skeletal muscle regeneration, and tissue-specific progenitors for various organ systems. MSCs (highlighted) are multipotent stromal cells that can be isolated from multiple tissue sources, including bone marrow, adipose tissue, dental pulp, synovium, and placenta. Among MSC sources, WJMSCs from umbilical cord tissue (double-highlighted with star) represent the primary focus of this review due to their accessibility from discarded perinatal tissue, non-invasive procurement, high proliferative capacity, and potent secretome profile suitable for cell-free regenerative applications in longevity-oriented precision medicine. Perinatal stem cells encompass multiple cell types derived from birth-associated tissues including umbilical cord blood, amniotic fluid, and placental membranes, offering advantages of low immunogenicity and ethical accessibility. This graphic is original and was created using the support of generative AI.

The broader landscape of stem cell types employed across regenerative medicine applications is shown in Figure 1, contextualizing the specific focus on MSC-derived secretome within this review. MSCs are multipotent cells that are found in multiple tissues, including bone marrow, adipose tissue, umbilical cord (UC) and Wharton’s jelly (WJ), with the capacity for self-renewal and differentiation into mesodermal lineages such as osteoblasts, chondrocytes and adipocytes. Beyond their differentiation potential, MSCs exert broad immunomodulatory, trophic and pro-regenerative effects through the secretion of bioactive molecules that influence surrounding cells and the extracellular matrix via paracrine and endocrine mechanisms [18,19,20]. These properties, together with their relative ease of isolation and expansion, have made MSCs central actors in regenerative medicine and a major focus of clinical translation efforts.

Among the available MSC sources, human umbilical cord WJ has emerged as a particularly attractive substrate for regenerative applications. WJMSCs can be obtained non-invasively from discarded perinatal tissue, avoiding donor-site morbidity and the many ethical concerns associated with other cell types. WJMSCs display a high proliferative capacity, robust clonogenicity and a secretory profile enriched for pro-angiogenic, anti-inflammatory and pro-regenerative factors compared with some adult MSC sources, although secretome composition is influenced by culture conditions [25,44]. Preclinical studies have shown that conditioned medium from WJMSCs enhances fibroblast proliferation and migration, accelerates re-epithelialization and improves granulation tissue quality in cutaneous wound models, supporting a potent paracrine contribution to tissue repair [24,25,27].

The term MSC secretome broadly encompasses the diverse array of bioactive molecules released by MSCs into their surrounding milieu, typically captured as conditioned medium under defined culture conditions. This includes soluble proteins such as growth factors, cytokines and chemokines; bioactive lipids; metabolites; extracellular matrix fragments; nucleic acids such as mRNA and microRNA; and vesicular as well as non-vesicular extracellular particles [18,22,23]. Proteomic and cytokine-profiling studies have identified recurring components across MSC secretomes, including interleukin-6 (IL-6), interleukin-8 (IL-8), monocyte chemoattractant protein-1 (MCP-1), vascular endothelial growth factor (VEGF), fibroblast growth factors (FGFs), transforming growth factor-beta (TGF-β) and tissue inhibitors of metalloproteinases (TIMPs), among others, although relative abundances vary by tissue source and culture modality [11,23]. In functional terms, these secreted factors collectively modulate inflammation, promote angiogenesis, support cell survival, influence matrix remodeling and, in some contexts, exert anti-fibrotic or anti-apoptotic effects [18,19].

A substantial portion of the MSC secretome is packaged within or associated with extracellular vesicles (EVs), a heterogeneous family of membrane-enclosed structures released by cells into the extracellular space [29,30,45]. EVs are generally categorized on the basis of biogenesis and size into exosomes, which originate from the endosomal system and are typically smaller than ~200 nm in diameter, and microvesicles or ectosomes, which bud directly from the plasma membrane and exhibit a broader size range [29,30,45]. Contemporary position statements from the International Society for Extracellular Vesicles emphasize that precise assignment to exosome or microvesicle subtypes requires rigorous demonstration of subcellular origin and that, in many studies, the term “extracellular vesicles” is more accurate than “exosomes” for describing isolated vesicle preparations [29,30,45]. Nonetheless, the term “exosomes” remains widely used in the regenerative medicine literature as a shorthand for small EVs obtained through standard ultracentrifugation or size-exclusion protocols.

MSC-derived EVs and exosomes transport a cargo of proteins, lipids and nucleic acids reflective of the parent cells and their activation state, enabling them to modulate a wide range of biological processes in recipient cells [46]. In preclinical models, MSC-EVs have been shown to recapitulate many of the therapeutic effects of their parent cells, including promoting angiogenesis, reducing inflammation, limiting apoptosis and enhancing tissue repair in settings such as myocardial infarction, acute kidney injury, liver injury and neurological issues [33,47,48,49,50,51]. In the context of skin and soft-tissue repair, exosome-enriched fractions from MSC cultures have been reported to accelerate wound closure, improve collagen organization and reduce scar formation, supporting their potential as cell-free regenerative agents [24,27,28]. These observations underpin the growing interest in exosome-based products as a more standardizable subset of the broader MSC secretome [22].

However, focusing solely on exosomes risks under-representing the full therapeutic complexity of the secretome. Studies comparing whole conditioned medium with isolated EVs suggest that non-vesicular components—including soluble growth factors, cytokines and chemokines—contribute significantly to the overall regenerative effect and may, in some contexts, act synergistically with vesicular cargo [18,28]. In other words, exosomes can be regarded as a potent but narrower slice of the broader secretome, which may or may not capture the full therapeutic synergy required in complex, multimorbid age-related conditions. This broader compositional diversity may be especially relevant in complex, multimorbid age-related conditions, where simultaneous modulation of multiple pathways—immune, vascular, matrix and neural—is likely required to achieve meaningful functional benefits [4,6,20].

In addition to proteins and extracellular vesicles, secretome also contains non-EV-associated low-molecular-weight metabolites and bioactive lipids that can modulate redox balance, energy metabolism and local inflammatory tone. Metabolomic profiling of conditioned media from MSC cultures has identified distinct amino-acid, carbohydrate and organic-acid signatures that differ between MSC sources and correlate with changes in glycolysis, oxidative phosphorylation and reactive oxygen species handling, suggesting that secreted metabolites may participate directly in the regulation of cell survival and stress responses in recipient tissues [52]. Complementary lipidomics studies of MSC secretome fractions demonstrate that specific lipid mediators, such as endocannabinoids and N-acylethanolamines, contribute to the anti-inflammatory and chondroprotective effects of conditioned media in osteoarthritis-like models, reinforcing the view that non-protein, non-vesicular components form an important part of the therapeutic payload [53]. Emerging data therefore support a model in which low-molecular-weight metabolites and bioactive lipids act together with vesicular cargo to underpin the pleiotropic effects of MSC secretome, particularly in metabolic and vascular aspects of aging, even though systematic characterization of these small-molecule fractions remains incomplete.

The concept of “stem cell therapy without the cells” arises directly from these insights. Rather than infusing large numbers of live cells and relying on uncertain in vivo survival and secretory activity, which are influenced by both cell-intrinsic properties and the recipient’s immune and health status, therapeutic strategies can instead administer concentrated secretome or defined EV-enriched fractions produced in controlled bioprocessing environments, where dosing can be more accurately standardized [20,21,22,23]. This approach holds several theoretical advantages: the same master cell bank can be used to generate multiple batches of product; manufacturing and quality control can focus on secretome composition and potency rather than cell viability; products can be stored and transported as off-the-shelf biologics; and the absence of viable cells may reduce certain safety and regulatory concerns, particularly for repeated dosing. For WJ-derived products, these advantages are amplified by the scalability and reproducibility of perinatal tissue sourcing, making WJMSC secretome an especially compelling candidate for integration into LPRM pathways [6,28].

**Figure 2:**
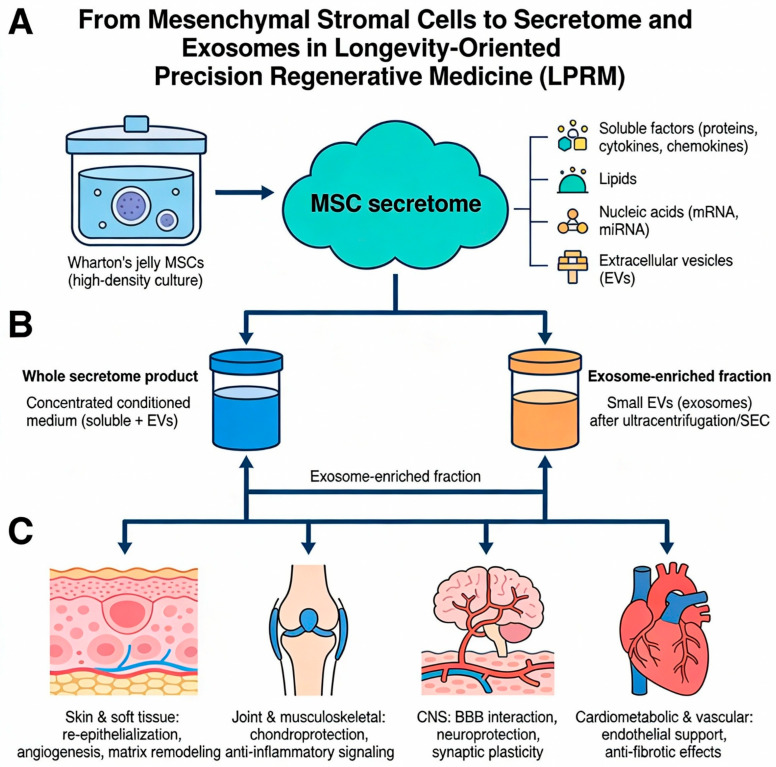
From MSCs to secretome and exosomes: mechanistic pathways and target systems in LPRM. (**A**) WJMSCs are expanded under high-density culture conditions and release a complex mixture of bioactive molecules into the culture medium (MSC secretome), including soluble proteins (growth factors, cytokines, chemokines and matrix-modifying enzymes), lipids, nucleic acids and extracellular vesicles (EVs) of different sizes. (**B**) The collected secretome can be used as a whole-secretome product after concentration by ultracentrifugation (UF) or tangential flow filtration (TFF) or further enriched using size-exclusion chromatography (SEC) to generate exosome-enriched fractions consisting predominantly of small EVs carrying selected protein and nucleic-acid cargo. (**C**) Both whole-secretome and exosome-enriched products act via pleiotropic paracrine mechanisms that are highly relevant to LPRM, including promotion of re-epithelialization, angiogenesis and matrix remodeling in skin and soft tissues; chondroprotection and anti-inflammatory activity in osteoarthritic joints; modulation of neuroinflammation, neuroprotection and synaptic plasticity within the central nervous system (in part via EV interaction with the blood–brain barrier); and support of endothelial function, anti-fibrotic signaling and metabolic regulation in cardiometabolic and vascular tissues. The schematic emphasizes how cell-free products translate MSC paracrine activity into standardized, off-the-shelf interventions suitable for iterative use within LPRM care pathways. This graphic is original and created using the support of generative AI.

Taken together, these biological foundations motivate a shift in focus from live MSC infusions toward optimized, standardized secretome- and exosome-based formulations that can be more readily integrated into LPRM programs, as schematically summarized in Figure 2.

### 3.1. Why Mesenchymal Stem Cells Are Preferred Secretome Sources

MSCs are widely used secretome producers because they combine robust self-renewal with broad immunomodulatory and trophic activity across diverse disease models and early-phase clinical trials. Clinical studies using bone-marrow-derived MSCs in indications such as graft-versus-host disease, Crohn’s disease, myocardial infarction and multiple sclerosis have repeatedly shown acceptable safety profiles together with signals of efficacy, supporting the notion that their paracrine output can modulate inflammation, promote tissue repair and improve functional outcomes in vivo [54]. These data underpin the use of MSCs as logical “biofactories” for cell-free products, since the same immunoregulatory and pro-regenerative factors that act in transplanted cells can be harvested as secretome for standardized administration.

Perinatal MSCs in particular exhibit high proliferative capacity, low baseline immunogenicity and a rich secretory profile compared with adult tissue-derived MSCs, making them attractive for scalable secretome and extracellular-vesicle production. Experimental work with perinatal MSCs from umbilical cord and related tissues shows enhanced expansion potential, reduced expression of costimulatory molecules and lower propensity for senescence-associated changes, suggesting that these cells can sustain consistent secretome output over extended culture without invasive donor procedures [23]. Together, these properties support the preferential use of MSCs—and especially perinatal MSCs—as master cells for generating off-the-shelf secretome and exosome preparations for LPRM.

### 3.2. Wharton’s Jelly MSCs in the Context of Longevity

WJMSCs are isolated from the mucoid connective tissue of discarded umbilical cords and therefore avoid donor-site morbidity and most ethical concerns while enabling the establishment of large-scale master cell banks from young, healthy tissue. Primary studies comparing perinatal sources demonstrate that WJMSCs display particularly high proliferation rates, low immunogenicity and stable phenotype during expansion, all of which are crucial for consistent, GMP-compatible production of paracrine products [55]. These attributes align with the needs of LPRM, where reproducible manufacturing and the possibility of repeated dosing in otherwise relatively healthy individuals are essential.

Functionally, WJMSC-derived secretome and extracellular vesicles have shown potent pro-regenerative and cytoprotective effects in preclinical and early translational work. Secretome produced from high-density WJMSC cultures accelerates wound closure, enhances re-epithelialization, and increases vascularization and granulation tissue quality in rodent wound models, illustrating its capacity to orchestrate complex tissue repair processes. Recent in vitro and ex vivo studies further indicate that WJMSC secretome can surpass bone marrow- and adipose-derived MSC secretomes in promoting fibroblast proliferation, extracellular-matrix remodeling and antioxidative responses relevant to skin aging and tissue regeneration [24]. These findings support the positioning of WJMSC secretome and exosomes as especially suitable effector products within LPRM while also providing a biological rationale for favoring WJMSC-based manufacturing platforms over adult MSC sources when scalability and product standardization are key. To synthesize these source-dependent differences, Table 1 compares key features of exosomes derived from various tissue sources in the context of LPRM applications.

## 4. Comparative Analysis: Cell Therapy vs. Secretome vs. Exosomes

### 4.1. Mechanistic Considerations

Mesenchymal stromal cell therapies were initially conceptualized as regenerative interventions based on engraftment and differentiation of transplanted cells into damaged tissues; however, a large body of evidence now supports a predominantly paracrine mode of action [18,23]. In multiple preclinical models, conditioned medium from MSCs, as well as isolated MSC-derived EVs, has been shown to reproduce many of the therapeutic effects of the parent cells, including modulation of inflammation, promotion of angiogenesis, enhancement of cell survival and stimulation of endogenous repair pathways [33,46]. These data underpin the view that the critical therapeutic “payload” resides in the secreted factors and vesicular cargo rather than in durable engraftment or transdifferentiation of the infused cells [18,23].

The whole MSC secretome represents the most comprehensive capture of this paracrine activity, incorporating soluble growth factors, cytokines, chemokines, lipids, nucleic acids and both vesicular and non-vesicular extracellular particles [18,19]. By contrast, exosome-focused preparations aim to isolate a more defined subset of small EVs enriched for specific proteins and nucleic acids, often with the intention of reducing complexity and simplifying characterization [29,48]. Comparative studies suggest that while exosomes recapitulate many of the immunomodulatory and pro-regenerative effects of the secretome, non-vesicular components can make substantial contributions in certain contexts, such as fibroblast migration, angiogenesis and matrix remodeling in wound-healing models [23,24]. In other words, exosomes can be regarded as a potent but narrower slice of the broader secretome, which may or may not capture the full therapeutic synergy required in complex, multimorbid age-related conditions [23,33].

From a mechanistic standpoint, secretome-based approaches offer a distinctive advantage over live cell therapies: they allow deliberate decoupling of paracrine function from in vivo cell behavior. In conventional MSC infusions, the magnitude, duration and localization of secretory activity depend on cell survival, homing, cellular expression dynamics and the surrounding microenvironment, all of which vary between patients and disease states and are difficult to control [56,57]. By contrast, secretome and exosome products can be manufactured under defined conditions that “pre-program” the paracrine profile—through choices of MSC source, culture density, inflammatory licensing and collection protocols—before being standardized into measurable doses [10,23]. This shift from in vivo dependence to ex vivo control is particularly attractive for LPRM, where reproducibility and fine-tuning of dose and schedule are essential [6,43].

### 4.2. Scalability, Manufacturing and Cost

Scalability remains one of the major constraints on the widespread adoption of MSC therapies, especially for preventive or wellness-oriented indications that might involve large numbers of relatively well individuals rather than small cohorts with advanced disease [12,31]. Manufacturing viable MSC products requires strict control of the cell source, expansion, passage number, cryopreservation for master and working banks and release criteria, with batch-to-batch variability and viability loss during storage and transport posing persistent challenges [16,31]. Additionally, producing large doses of cells for repeated systemic administration can be resource-intensive, as each treatment course consumes a substantial number of cells from a given donor or cell bank [12,58].

Secretome-based approaches substantially alter this equation. Because the same population of MSCs can be used to generate multiple batches of conditioned medium over time, secretome production effectively “collects the eggs rather than sacrificing the chicken,” maximizing the output from each carefully characterized master cell bank [18,23]. High-density culture systems, including bioreactors and modern, chemically defined media, allow further amplification of secretome yield per unit of culture surface or volume, and downstream processing steps such as concentration and lyophilization facilitate the creation of storable, ready-to-use formulations [18,58]. This inherently higher productivity per donor and per culture run translates into more favorable unit economics, especially when secretome is used in indications requiring repeated or maintenance dosing—precisely the pattern anticipated in longevity- and healthspan-oriented programs.

Exosome-focused products occupy an intermediate position. On one hand, they are derived from the same cultures and thus benefit from the underlying scalability of secretome production; on the other, additional isolation and purification steps (such as ultracentrifugation, density gradients or size-exclusion chromatography) add complexity, cost and potential for product loss [33,59]. While these extra steps may be justified for tightly targeted indications where a more defined EV product is advantageous, they can limit the cost-effectiveness of exosomes for broad, preventive applications compared with whole secretome, which can be processed more simply while retaining a wide array of bioactive components [18,33]. Overall, when considering scalability, manufacturing efficiency and cost, secretome-based products appear particularly well-suited to large-scale deployment in LPRM.

### 4.3. Cellular Aging, Passage Number and Master Cell Banks

Secretome and exosome products rely on well-characterized master and working cell banks because prolonged ex vivo expansion of MSCs leads to replicative senescence, altered paracrine profiles and a progressive loss of therapeutic potential. Serial-passaging studies in human MSCs show that increasing passage number is accompanied by mitochondrial dysfunction, elevated reactive oxygen species and senescence-associated gene expression—changes that correlate with reduced proliferative capacity and impaired differentiation and paracrine activity [60]. Likewise, experiments comparing MSC secretome from early versus later passages demonstrate passage-dependent shifts in the abundance of pro-angiogenic factors and diminished ability to stimulate angiogenic signaling at higher passages, supporting the practice of restricting passage numbers when generating clinical-grade secretome and EV products [61].

Standard practice therefore couples passage limits with assays that monitor senescence markers and functional potency to ensure that product batches reflect a youthful, stable phenotype of the master cells. Assurance of therapeutic activity across lots further requires predefined quality attributes, such as EV size distribution and cargo characteristics, together with functional in vitro readouts like fibroblast migration and endothelial tube-formation assays that have been shown to respond in a dose-dependent manner to MSC-derived exosomes [62]. By combining these potency-linked assays with tight control of culture conditions and harvest timing, manufacturers can reduce batch-to-batch variability and maintain consistent bioactivity of secretome and exosome preparations over the lifespan of a master cell bank.

### 4.4. Safety and Tolerability

Safety considerations are paramount when regenerative interventions are proposed for individuals who may be in earlier stages of disease or seeking to extend their healthspan. Systemic administration of high-dose live MSCs, especially allogeneic cells, raises several theoretical and observed safety concerns, including transient microvascular obstruction, pro-thrombotic effects, immunogenic reactions and, in rare cases, ectopic tissue formation or pro-tumorigenic influences in susceptible settings [13]. While many clinical trials have reported acceptable short-term safety profiles and there has been over 20 years of practical track record of using various types of stem cells in clinics across Asia, largely in Thailand, these risks still necessitate rigorous pre-treatment diagnostic profiling, cautious dosing, prolonged infusion times and close clinical monitoring, potentially limiting feasibility for repeated, outpatient regimens in wellness-oriented contexts [12].

For perspective, autologous options, including platelet-rich plasma (PRP) and adipose-derived cell preparations remain in use, mostly for facial rejuvenation, but reported benefits are heterogeneous and often based on subjective improvement rather than robust, long-term outcome data [63,64]. At the same time, market demand in the region has shifted towards allogeneic products sourced from younger tissues (prominently umbilical cord-derived MSCs or their conditioned media, being a less sophisticated version of secretome), which are considered to have stronger outcomes. Looking further ahead, the growing practice of banking umbilical cord blood and perinatal tissues means that some individuals may eventually access autologous “young” umbilical cord-derived MSCs or their secretome either when sick or injured or in later life, adding a further layer of complexity to the autologous–allogeneic distinction [65,66].

Secretome-based products, by eliminating viable cells, remove the risk associated with uncontrolled in vivo cell behavior, including proliferation and long-term persistence. Adding to this challenge is the lack of ability to track and trace live MSCs once implanted. EV and biomolecule safety profiles are instead governed by factors such as the immunogenicity of the proteins and vesicle cargo, the presence of residual process-related impurities and the potential for off-target bioactivity of specific cytokines or growth factors at supraphysiological concentrations [33]. Early human experience with MSC-derived secretome and exosomes, including both formal trials and physician-led clinical use in diabetes, chronic wounds and neurodegenerative conditions, suggests that these products are generally well tolerated when appropriately manufactured and administered, though rigorous, long-term pharmacovigilance data are still emerging [24,67,68].

From a practical perspective, the reduced risk profile of acellular products supports shorter infusion times, simpler monitoring requirements and the feasibility of repeat dosing, which are all critical for integration into longitudinal, preventive care pathways. Moreover, the absence of viable cells simplifies donor screening and viral safety strategies, as inactivation steps not compatible with live cells can be used during processing, potentially enhancing overall product safety [18]. Exosome-focused products may offer additional safety advantages in specific contexts—such as reduced protein content and a narrower cargo profile—though the clinical implications of these differences are still being elucidated and may vary by indication [33]. In aggregate, the safety and tolerability profile of secretome and exosome products appears better aligned with the needs of longevity-focused and preventive applications than conventional high-dose cell therapies.

### 4.5. Standardization of Secretome and Exosome Production

While no universally accepted standard for medical-grade exosome isolation exists, several scalable approaches have been validated, combining high-density culture with methods such as tangential flow filtration, ultrafiltration and size-exclusion chromatography. Microcarrier-based 3D culture of MSCs coupled with tangential flow filtration increases exosome yield and maintains biological activity compared with conventional 2D culture and ultracentrifugation, illustrating how process choice directly affects both particle recovery and functional potency [69]. Size-exclusion chromatography and related fast liquid-chromatography variants can efficiently enrich small extracellular vesicles with preserved size distribution and reduced protein contaminants, supporting their use as downstream steps after initial concentration of conditioned media [70,71]. In parallel, pilot GMP-compliant workflows for freeze-dried MSC secretome demonstrate that whole secretome can be concentrated by ultrafiltration and lyophilized into standardized batches suitable for clinical use, provided that isolation and formulation parameters are tightly controlled [72].

For clinical programs, a pragmatic pathway is to first generate and concentrate whole secretome under closed or semi-closed conditions using standardized, xenofree media, and then optionally perform downstream enrichment into small-EV (exosome-enriched) fractions when a narrower cargo profile is desired. Across these workflows, orthogonal analytics—including nanoparticle tracking or equivalent methods for particle size/number, protein quantification and functional in vitro assays—are used to define lot-release criteria and monitor batch consistency. By integrating scalable concentration methods (e.g., tangential flow filtration or ultrafiltration), gentle EV-enrichment techniques such as size-exclusion chromatography, and in-process controls, a realistic standardization strategy that is compatible with current regulatory expectations for biologics and advanced therapy medicinal products (ATMP) can be approached.

### 4.6. Secretome Versus Exosome-Enriched Formulations

Whole secretome captures both vesicular and non-vesicular components and may be preferable where broad immunomodulation and matrix remodeling are desired. Comparative in vitro and in vivo studies show that unfractionated MSC conditioned media can accelerate wound closure, enhance re-epithelialization and improve barrier integrity, effects that reflect the combined action of soluble cytokines, growth factors, metabolites and extracellular vesicles. In osteoarthritis-like models, whole secretome has been reported to exert chondroprotective effects linked not only to EV cargo but also to specific bioactive lipids and soluble mediators, supporting the view that retaining non-vesicular fractions can be advantageous when complex, multifactorial tissue responses are required [24,73].

By contrast, exosome-enriched or small-EV formulations offer a more defined but narrower cargo that is particularly attractive for targeted applications and for engineering as drug-delivery vehicles [74]. MSC-derived exosomes isolated and concentrated by scalable processes induce dose-dependent proliferation, migration and tube formation in endothelial and epithelial cells, and can efficiently deliver nucleic acids or other therapeutic payloads to target cells in vitro and in animal models [33,51]. In LPRM contexts that anticipate repeated outpatient dosing, the choice between administering whole-secretome or exosome-enriched products therefore reflects a trade-off between compositional complexity, manufacturing cost and regulatory positioning: whole secretome favors breadth of bioactivity and simpler upstream processing, whereas exosome-focused products favor tighter characterization and therefore regulation, as well as easier adaptation to targeted or cargo-loaded strategies.

### 4.7. Practical Aspects of Administration

Practical considerations around the preparation, route and duration of administration have major implications for how regenerative therapies can be integrated into routine clinical and LPRM practice. Intravenous MSC infusions often involve relatively large volumes of cell suspension delivered over extended periods—frequently one to several hours—to minimize risks such as microvascular obstruction and to preserve cell viability during administration [12]. Studies examining MSC viability during infusion indicate that cell survival can decline substantially over time depending on the suspension medium and infusion duration, underscoring the tension between safety-driven slow infusions and the need to deliver viable cells efficiently. These logistical constraints typically necessitate dedicated clinical settings, continuous monitoring and physician oversight, which may be acceptable for severe disease but are less compatible with high-throughput preventive or wellness programs [75,76].

By contrast, secretome- and exosome-based products can be formulated as stable solutions or lyophilized powders that are reconstituted immediately prior to use, with dosing defined by concentration of key components rather than cell counts. Intravenous administration of such acellular products can generally be completed over significantly shorter periods—often within 30–60 min for typical infusion volumes—without concern for real-time cell viability and with a reduced need for intensive monitoring once early tolerability has been established [68]. In local applications, including intra-articular injections for arthropathy or topical application for wound healing, secretome can be delivered in concentrated, small-volume formats such as gels, hydrogels or depot formulations, further simplifying logistics and enhancing patient comfort [77,78]. Overall, these practical advantages make secretome-based interventions more readily adaptable to the outpatient, iterative treatment models typical of LPRM.

### 4.8. Central Nervous System and Blood–Brain Barrier Considerations

The central nervous system (CNS) poses a unique challenge for regenerative interventions because of the restrictive properties of the blood–brain barrier (BBB), which limits the passage of many therapeutics from the circulation into neural tissue [79,80]. While some studies have suggested that intravenously administered MSCs can home in on areas of brain injury, the proportion of cells that reach the CNS is typically small, and a substantial fraction may become trapped in non-target organs such as the lungs, liver and spleen [41]. Direct intracerebral or intrathecal delivery of cells can increase local exposure but entails invasive procedures and potential long-term uncertainty regarding cell persistence, behavior and immunological interactions within the CNS milieu [41,81].

EVs, including exosomes derived from MSCs, have attracted intense interest as potentially superior vehicles for CNS-directed regenerative therapy. EVs are nanoscale, lipid-bilayer particles that can cross biological barriers, including the BBB, via active transcytotic mechanisms; several studies demonstrate that both endogenous and engineered EVs can traverse brain endothelial cells and deliver cargo to neurons, glia and other CNS cell types [41]. In preclinical models of ischemic stroke, MSC-derived exosomes have been shown to promote neurogenesis, angiogenesis, synaptic plasticity and functional recovery, in some cases achieving outcomes comparable to or better than whole-cell therapy. Similar findings have been reported in animal models of traumatic brain injury and neurodegenerative diseases, where exosome-mediated modulation of inflammation, oxidative stress and apoptosis appears to underpin neuroprotective effects [47,81].

Secretome-based products that include both EV and non-vesicular components may offer additional advantages for CNS indications. Soluble neurotrophic factors, anti-inflammatory cytokines and matrix-modifying enzymes present in the secretome can act synergistically with EV cargo, and there is emerging evidence that systemically administered secretome can influence CNS function indirectly by modulating peripheral immune and vascular pathways that communicate with the brain [46]. Early clinical experiences and initial trials with MSC-derived exosomes in conditions such as Alzheimer’s disease suggest that intranasal or intravenous delivery of EV-rich preparations is feasible and may yield cognitive and functional benefits, although robust, large-scale data remain limited [47,68]. Taken together, the capacity of EV-containing secretome to engage the CNS through both direct BBB crossing and systemic signaling pathways provides a mechanistic rationale for prioritizing cell-free approaches in neurodegenerative and cognitive-decline indications within LPRM.

### 4.9. Clinical and Preclinical Outcomes in Longevity-Relevant Conditions

Evidence for MSCs’, secretome and exosomes’ positive regenerative effects spans a wide spectrum of conditions relevant to aging, including musculoskeletal degeneration, type 2 diabetes, chronic wounds, cardiometabolic disease and CNS disorders, though the depth of clinical data varies considerably between indications and product types [46]. In osteoarthritis, multiple clinical trials have evaluated intra-articular MSC therapies, demonstrating improvements in pain and function and, in some cases, structural cartilage benefits. More recently, pilot studies using MSC-derived conditioned medium or secretome, alone or in combination with platelet-derived products, have reported encouraging signals of symptom relief and structural benefit, albeit in small cohorts. These findings support the hypothesis that a substantial proportion of MSC benefit in joint disease can be recapitulated by cell-free products, with potential advantages in standardization and repeatability [39,77,82].

**Figure 3:**
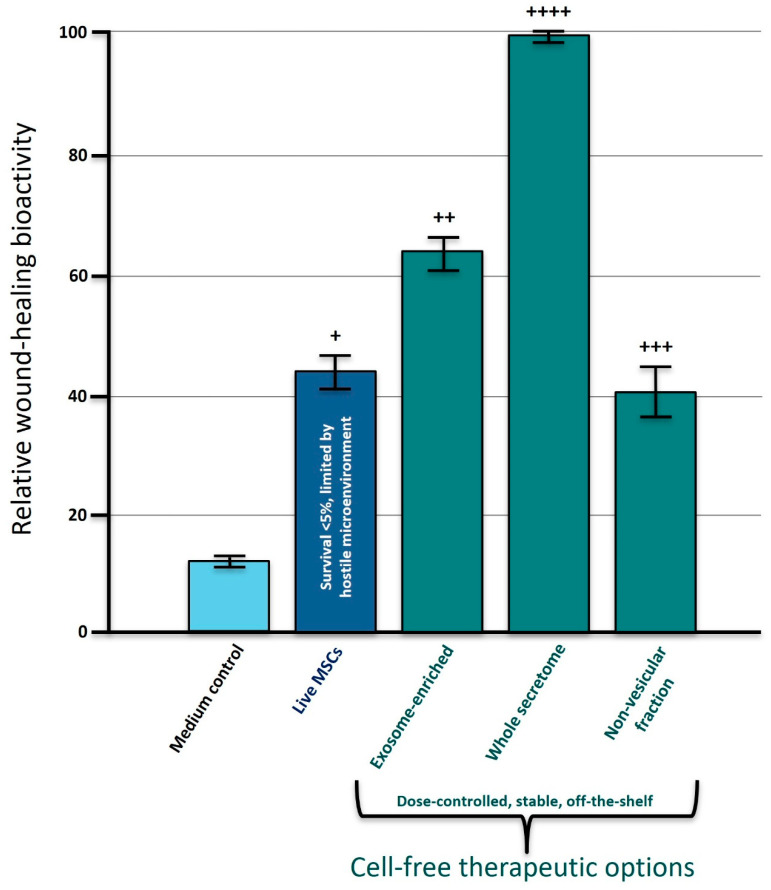
Superior pro-regenerative activity of secretome compared to live cells in wound healing. A qualitative summary of the relative wound-repair bioactivity of different MSC-derived therapeutic approaches based on in vitro and in vivo wound-healing models [21,24,27,28,84]. It depicts an ordinal scale of wound-repair activity, demonstrating that (i) live MSCs, while exerting beneficial effects beyond baseline (medium controls), face significant therapeutic constraints in vivo. These include extremely poor cell survival rates (<5% retention/survival at wound sites within days), vulnerability to the hostile wound microenvironment (hypoxia, inflammation, reactive oxygen species, absence of trophic support), limited homing efficiency, and challenges in standardized dosing [85,86]. The paracrine output of transplanted MSCs is therefore substantially restricted by cell viability and microenvironmental barriers. (ii) Whole MSC secretome demonstrates the highest pro-healing bioactivity and is set here at 100% for comparative analysis. This cell-free approach overcomes cell survival limitations and can be precisely dosed, concentrated through validated methods (ultrafiltration, lyophilization), stored stably (months at ambient temperatures, years lyophilized and indefinitely at −80 °C), and deployed as an off-the-shelf therapeutic. Concentrated secretome preparations consistently accelerate scratch closure, re-epithelialization, granulation tissue maturation, and angiogenesis. The secretome’s superior performance reflects the fact that it delivers the complete paracrine payload without the constraints of cellular fragility, enabling therapeutic optimization considered next to impossible with living cells. (iii) Exosome-enriched secretome fractions and non-vesicular soluble factor fractions, when separated, each retain substantial regenerative activity. These findings indicate that multiple components within the secretome contribute to wound healing. It is well established that exosomes and other small extracellular vesicles appear to mediate a large share of the wound-healing activity of the MSC secretome, while soluble factors and larger vesicles provide additional, complementary therapeutic effects [87,88,89]. Qualitative indication of relative wound-healing bioactivity and stability is denoted by the number of ‘+’ symbols: + = low, ++ = moderate, +++ = high, ++++ = very high.

Chronic, non-healing wounds represent one of the clearest exemplars of secretome efficacy in a condition tightly linked to aging and chronic disease. WJMSC-derived secretome has been shown to greatly accelerate wound closure, enhance re-epithelialization and modulate scar formation in both in vitro assays (scratch-closure assays, three-dimensional skin equivalents) and animal models (rat acute wound models) [24]. These preclinical data are complemented by emerging clinical studies using secretome- or exosome-based formulations—such as secretome-containing hydrogels and topical EV preparations—in difficult-to-heal ulcers, which collectively suggest high rates of wound closure and improved tissue quality with acceptable safety profiles [83]. The relative contributions of live MSCs, whole secretome and exosome-enriched fractions to wound-repair activity are illustrated conceptually in Figure 3.

Similar patterns are beginning to emerge in early-phase investigations of secretome and exosomes in neurodegenerative disease and age-related cognitive decline, where preliminary signals of cognitive and functional improvement are now being explored in controlled trials [47,68]. Taken together, these clinical and translational data reinforce the central argument of this review: that cell-free formulations of MSC secretome and EVs can capture much of the therapeutic value of traditional MSC therapy while offering superior scalability, practicality and alignment with the needs of longevity-oriented precision regenerative medicine. The key similarities and differences between live MSC therapies, whole secretome and exosome-enriched products across these dimensions are summarized in Table 2.

## 5. Clinical and Translational Evidence in Longevity-Relevant Conditions

There is considerable evidence emerging that MSCs, secretome and exosomes provide a versatile intervention concept in regenerative medicine applications, spanning a broad range of conditions that are tightly coupled to aging trajectories (such as musculoskeletal degeneration, diabetes and related ailments such as ulcers and chronic wounds, cardiometabolic disease and central nervous system (CNS) disorders, although the availability of clinical data varies significantly between morbidities and products [18,46]. In general, live MSC therapies are supported by more extensive late-phase human data, whereas secretome- and exosome-based products are currently underpinned by a combination of robust preclinical findings, early clinical experiences and a small number of formal trials.

### 5.1. Osteoarthritis and Degenerative Joint Disease

Osteoarthritis (OA) is one of the most prevalent age-associated conditions and a major driver of pain, disability and healthcare utilization. Intra-articular MSC therapies have been evaluated in multiple clinical studies, with several trials reporting improvements in pain and function—often measured by WOMAC and VAS scores—as well as radiological or MRI-based signals of structural benefit. These data have contributed to regulatory approvals for MSC products targeting musculoskeletal indications in selected jurisdictions and demonstrate that paracrine-driven regenerative strategies can meaningfully modify joint homeostasis in at least a subset of patients [39,82].

Secretome-based approaches for OA are at an earlier stage but are conceptually and mechanistically grounded in this experience. Preclinical models show that MSC-derived conditioned medium can reduce synovial inflammation, modulate catabolic cytokine profiles and support chondrocyte survival and matrix synthesis, sometimes achieving effects comparable to live cells [46]. In an equine model of joint inflammation, intra-articular administration of an allogeneic MSC secretome preparation reduced synovial inflammation and improved clinical parameters to a degree similar to that observed with live MSCs, reinforcing the notion that the therapeutic payload is largely paracrine and can be captured in acellular formulations [77]. Small pilot studies and physician-led series in humans using MSC-conditioned medium, often combined with platelet-derived products, have reported symptomatic improvements in OA, though patient numbers remain modest and standardized outcome measures are inconsistently applied.

### 5.2. Chronic Wounds and Diabetic Foot Ulcers

Chronic wounds, including diabetic foot ulcers, venous leg ulcers and pressure ulcers, represent a major source of morbidity, reduced quality of life and healthcare cost in older adults and individuals with multimorbidity. Among all longevity-relevant indications, wound healing offers some of the clearest preclinical evidence and emerging clinical support for MSC-derived secretome [24,84]. WJMSC-conditioned medium has consistently been shown to accelerate wound closure, enhance re-epithelialization, improve granulation tissue quality and modulate scar formation in in vitro scratch-assay systems, three-dimensional skin equivalents and rodent wound models [24,27].

Early human work has begun to translate these findings. In patients with diabetic foot ulcers, a topical gel containing human cord-derived MSC secretome has been associated with significantly greater reductions in wound area and faster time to closure compared with base gel controls, alongside increased local IL-10 expression and attenuation of NF-κB-related inflammatory signaling [83]. A systematic review and meta-analysis of in vivo secretome studies in diabetic wounds further supports its beneficial effects on angiogenesis, epithelialization, collagen organization and inflammatory resolution, although it highlights substantial heterogeneity in cell source, formulation and dosing regimens. Parallel efforts using exosome-enriched preparations within hydrogels and advanced dressings similarly demonstrate accelerated healing in diabetic and ischemic wound models, suggesting that both vesicular and non-vesicular components of the secretome can be harnessed for clinically meaningful tissue repair [33,46].

### 5.3. Type 2 Diabetes Mellitus and Metabolic Dysfunction

Type 2 diabetes mellitus (T2DM), characterized by progressive β-cell dysfunction, insulin resistance and chronic low-grade inflammation, represents one of the most prevalent age-associated metabolic disorders and a major driver of cardiovascular, renal and neurovascular complications [90]. Traditional pharmacologic therapies can achieve glycemic control but often fail to halt disease progression or reverse underlying pathophysiology, creating strong rationale for regenerative strategies that address both β-cell loss and peripheral insulin resistance simultaneously [91].

Preclinical evidence indicates that MSC-derived exosomes and secretome exert pleiotropic effects across multiple pathogenic mechanisms in T2DM. In streptozotocin (STZ)-induced and high-fat-diet models, administration of human umbilical cord MSC-derived exosomes (hUCMSC-Exos) has been shown to reduce blood glucose levels, improve glucose tolerance and insulin sensitivity, and restore pancreatic β-cell mass and insulin secretion [92,93]. Mechanistically, MSC-EVs promote β-cell proliferation and inhibit apoptosis through Pdx1-dependent signaling pathways, reverse peripheral insulin resistance by restoring phosphorylation of insulin receptor substrate-1 (IRS-1) and Akt, increase GLUT4 expression and membrane translocation in skeletal muscle, and enhance hepatic glycogen storage [92,94]. In parallel, MSC-EVs modulate the inflammatory milieu by reprogramming M1 (pro-inflammatory) macrophages toward the M2 (anti-inflammatory) phenotype and enhancing regulatory T-cell function, contributing to improved systemic metabolic homeostasis [95,96].

Early clinical experience with MSC therapies in T2DM patients, predominantly from Asian centers, supports translational feasibility. Multiple phase I/II trials using intravenous or intrapancreatic infusions of umbilical cord-derived MSCs have reported significant reductions in HbA1c, improvements in fasting and stimulated C-peptide levels, and decreased insulin requirements over 6- to 12-month follow-up periods, with acceptable safety profiles [97,98]. Post hoc analyses suggest that male patients with preserved baseline C-peptide reserve may be more likely to respond clinically to UC-MSC therapy, highlighting the potential for biomarker-guided patient selection [98]. More recently, a phase I/II clinical trial registered in Indonesia is evaluating MSC-derived exosomes specifically for glycemic control in T2DM, representing one of the first formal assessments of acellular EV products in metabolic disease [67]. Additionally, a controlled rat study using hypoxia-preconditioned MSC secretome demonstrated dose-dependent reductions in Homeostasis Model Assessment-Insulin Resistance (HOMA-IR, a clinical parameter for insulin resistance), preservation of pancreatic islet architecture and suppression of inflammatory markers, further supporting the therapeutic potential of cell-free secretome formulations in T2DM [99].

Within the LPRM framework, secretome-based interventions for T2DM and prediabetes could be conceptualized as part of multi-modal metabolic optimization programs that integrate lifestyle modification, pharmacotherapy and biomarker-guided monitoring. For individuals with early metabolic dysfunction—characterized by insulin resistance, elevated inflammatory markers and declining β-cell reserve but not yet meeting full diagnostic criteria for T2DM-intermittent courses of MSC-derived exosomes or secretome, delivered intravenously or via other systemic routes, might be deployed to delay progression, preserve β-cell function and reduce long-term complication risk. As with other LPRM applications, formal clinical trial data, standardized product characterization and transparent communication about evidence levels will be essential to integrate these approaches into preventive and early-intervention pathways.

### 5.4. Neurodegenerative and CNS Indications

Neurodegenerative diseases such as Alzheimer’s and Parkinson’s disease, as well as acquired CNS injuries like stroke and traumatic brain injury (TBI), are among the most feared consequences of aging and a major target for LPRM. Preclinical studies indicate that MSC-derived exosomes can cross or modulate the blood–brain barrier, deliver neurotrophic and anti-inflammatory cargo to neural and glial cells and support neurogenesis, synaptic plasticity and angiogenesis in models of stroke, TBI and Parkinsonian neurodegeneration [41,47]. In these models, exosome therapy often matches or exceeds the functional improvements achieved with live MSC administration, while avoiding concerns about long-term cell engraftment and ectopic proliferation.

The first-in-human data in Alzheimer’s disease is cautiously encouraging. In a phase I/II study of intranasally delivered allogeneic adipose MSC-derived exosomes in patients with mild-to-moderate Alzheimer’s disease, treatment was well tolerated across dose cohorts, with no serious adverse events reported [68,100]. Exploratory efficacy analyses suggested that medium-dose exosome administration was associated with stabilization or improvement in cognitive scores (e.g., ADAS-cog and MoCA) and reduced hippocampal volume loss over 36 weeks, although the small sample size and open-label design mandate cautious interpretation [68,101]. Complementary systematic reviews of intranasal administration of stem cell derivatives in AD models report consistent improvements in learning, memory and neuropathological markers, bolstering the rationale for larger, controlled human trials of exosome-enriched secretome in neurodegenerative disease [102,103].

### 5.5. Cardiometabolic and Systematic Indications

Compared with musculoskeletal, wound and CNS applications, cardiometabolic indications for MSC-derived secretome and exosomes remain largely at the preclinical stage. Nonetheless, a substantial body of work indicates that secretome components can ameliorate endothelial dysfunction, reduce oxidative stress, promote angiogenesis and modulate inflammatory and fibrotic signaling in models of ischemic heart disease, peripheral artery disease and metabolic syndrome [46,50]. Many of the mechanistic themes observed in diabetic wound-healing—such as exosomal delivery of pro-angiogenic miRNAs, modulation of macrophage polarization and enhancement of microvascular integrity—are highly relevant to cardiometabolic organ systems and suggest that secretome-based approaches may eventually find a place in preventive or adjunctive strategies targeting vascular aging and cardiometabolic risk [46,50]. For now, however, translating these insights into LPRM practice will require carefully designed early-phase human studies with clear safety and efficacy endpoints.

## 6. Regulatory and Regional Perspectives with a Southeast Asian Focus

The regulatory environment for regenerative products—both cell-based and cell-free—remains heterogeneous across jurisdictions, reflecting different risk tolerances, scientific capacities and policy priorities [16]. In regions such as the European Union, United States and Japan, most MSC therapies and their derivatives are regulated as advanced therapy medicinal products or similar categories, facing stringent quality, safety and efficacy requirements and, in many cases, centralized marketing authorization procedures. These frameworks have enabled the approval of a limited number of MSC-based products for indications such as steroid-refractory graft-versus-host disease and complex perianal fistulas, but they also impose substantial development costs and timelines, constraining access outside formal trials and specialized centers [7,9,16].

In Southeast Asia, and particularly in Thailand, regulatory approaches to cell and gene therapies have evolved rapidly over the past two decades. Early stem cell research initiatives in Thailand date back to the mid-1990s, with the first national policies and guidelines emerging in the 2000s to govern clinical use and research. By 2009, the Thai Food and Drug Administration and the Medical Council of Thailand had introduced specific guidance for stem cell treatments, focusing on ethical oversight, scientific justification and limitations on direct-to-consumer marketing of unproven therapies [31]. More recently, Thailand has moved toward adopting an ATMP-like framework, with classification pathways and regulatory requirements that mirror those of more established regulators, while still allowing some flexibility for hospital-based and investigator-initiated applications.

Thailand’s position as a major global medical-tourism hub adds a further layer of complexity. The country attracts large numbers of international patients and generates substantial revenue from medical tourism, with projections indicating continued growth driven by demographic aging and demand for high-quality, cost-effective care [32]. Within this ecosystem, regenerative interventions—including live MSC therapies and, more recently, secretome- and exosome-based products—have emerged as differentiating offerings in integrated wellness and longevity programs. This creates both opportunity and risk: on one hand, Thailand can serve as an innovation testbed and data source for LPRM models; on the other, it raises concerns about variable evidence standards, cross-border regulatory arbitrage, efficiency of data collection and the marketing of interventions whose clinical benefits are still being established [31,104].

While Thailand and Southeast Asia exemplify rapidly evolving regenerative-longevity hubs, similar trends toward integrating cell-free regenerative products into preventive care are emerging in other regions, including North America, parts of Europe and East Asia, albeit under diverse regulatory frameworks. We briefly situate the Thai experience within this broader global landscape to provide context for clinicians, biomedical entrepreneurs and policymakers in other jurisdictions.

For cell-free products, regulatory classification remains a key challenge. Complex, multi-component MSC secretome preparations may be viewed as biologics or combination products rather than conventional small molecules or single-target biologics, complicating the design of potency assays and release criteria [18]. Exosome-enriched products, with a more tightly defined vesicular cargo and standardized EV characterization under guidelines such as MISEV2023, may be somewhat more straightforward to align with existing biologics frameworks, but questions remain with regard to their long-term safety, immunogenicity and manufacturing scalability [29,105]. In Southeast Asia, regulators are beginning to grapple with these issues, but clear, harmonized pathways for secretome and exosomes are still under development, underscoring the need for ongoing dialogue between scientists, clinicians, regulators and industry.

## 7. The Preventive Longevity Patient Journey

**Figure 4:**
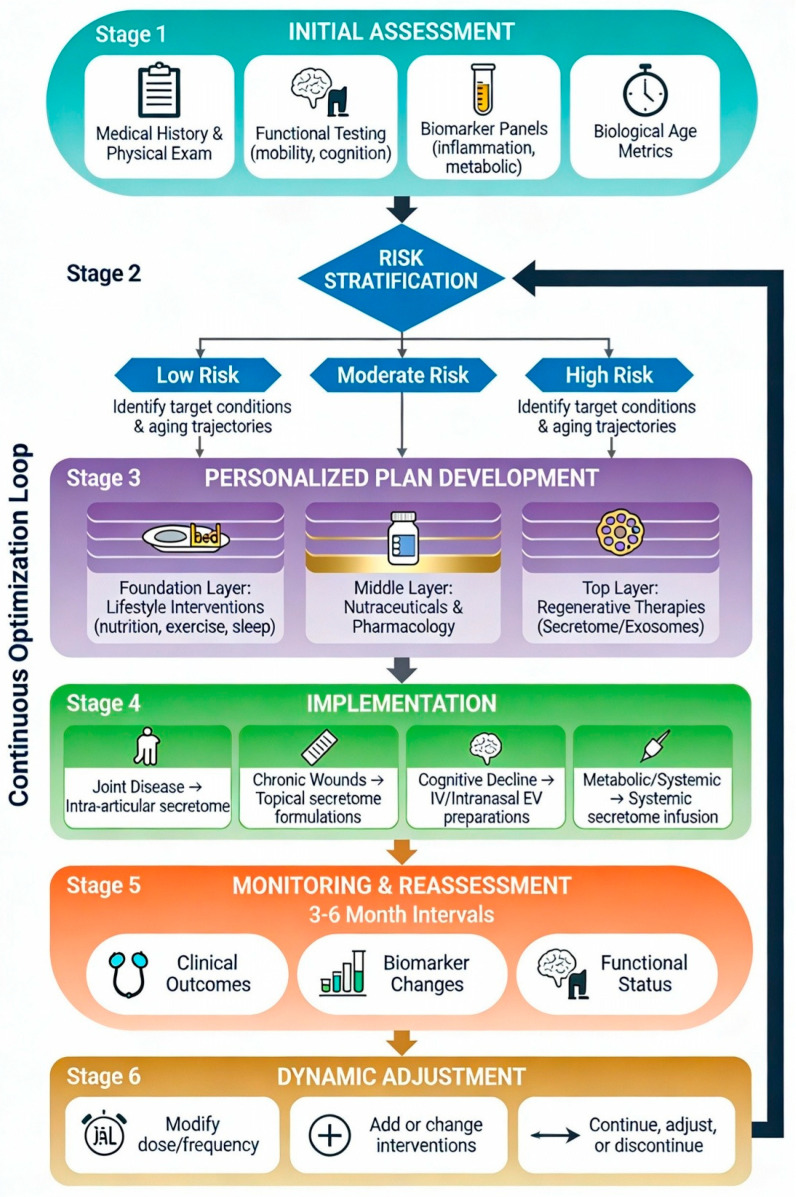
Concept for integrated patient journey incorporating cell-free interventions. The LPRM pathway begins with a comprehensive assessment including clinical evaluation, functional testing, advanced biomarker profiling, and biological age metrics to establish baseline status and identify early markers of age-related decline. Patients are stratified into risk categories based on objective measures, and personalized multi-modal plans are developed that layer interventions from foundational lifestyle modifications (nutrition, exercise, sleep optimization, and stress management) through targeted nutraceutical and pharmacological support to strategic regenerative therapies. Cell-free MSC secretome- and exosome-based products can be condition-specifically integrated: intra-articular administration for osteoarthritic joint disease and musculoskeletal degeneration, topical or local application for wounds and soft-tissue repair, intravenous or intranasal delivery for neurodegenerative concerns and cognitive preservation, and systemic infusion for broader metabolic and inflammatory modulation. The pathway emphasizes iterative monitoring at defined intervals (typically 3–6 months), with reassessment of clinical outcomes, functional status, and biomarker trajectories enabling dynamic adjustment of therapeutic dose, frequency, and modality selection. This closed-loop optimization model ensures that regenerative interventions remain anchored in measurable benefit and evidence-based practice, distinguishing precision longevity medicine from speculative anti-aging approaches. The framework is particularly well-suited to integrated medical-wellness and longevity clinic environments where comprehensive diagnostic services, lifestyle interventions, and advanced regenerative therapies can be coordinated longitudinally for both preventive and early-intervention applications.

Within the LPRM framework, regenerative interventions such as MSC-derived secretome and exosomes are most effectively conceptualized not as standalone procedures but as components of a broader, longitudinal care pathway aimed at preserving function and delaying the onset or progression of chronic disease [43]. Contemporary models for healthy-longevity clinics emphasize a structured, iterative patient journey that integrates comprehensive assessment, personalized planning, multi-modal interventions and regular monitoring and recalibration [6] (see also Figure 4).

A typical journey begins with an initial assessment phase, during which clinicians collect a detailed medical and family history, lifestyle and psychosocial data, and perform a focused physical examination, functional tests (e.g., gait speed, grip strength, balance), body composition analysis and targeted cardiometabolic, inflammatory and hormonal biomarker panels [6]. Where feasible, this may be augmented by measures of biological age or frailty indices and, in some settings, multi-omic or advanced imaging assessments. Using these data, patients are stratified into risk profiles, and a personalized plan is developed that prioritizes foundational lifestyle changes—nutrition, physical activity, sleep and stress management—supported by evidence-based pharmacologic and nutraceutical interventions as indicated [6].

Within this scaffold, regenerative cell-free products such as WJMSC secretome or MSC-derived exosomes can be layered in at defined points, guided by both clinical status and biomarker trajectories. For example, in individuals with symptomatic but non-end-stage osteoarthritic joint disease, intra-articular or peri-articular use of secretome-based formulations might be integrated into a broader musculoskeletal rehabilitation program focused on strength, mobility and weight management. In patients with chronic or recurrent wounds, topical or locally injected secretome preparations could be deployed alongside advanced wound care, glycemic control and vascular assessment to accelerate healing and reduce recurrence risk [83,84]. For older adults with early cognitive complaints or elevated neurodegenerative risk markers, carefully monitored courses of EV-rich secretome, delivered intravenously or intranasally, might be embedded within multi-modal cognitive-health programs that also address vascular risk factors, sleep, physical activity and social engagement [47,68].

Follow-up appointments at defined intervals then reassess clinical outcomes, functional status and selected biomarkers, enabling clinicians to adjust both foundational and regenerative components of the plan. Over time, this iterative process allows for dynamic adjustments of secretome-based interventions—modifying the dose, frequency or route—and ensures that their use remains anchored in measurable benefit rather than anecdote [6,106]. Crucially, clear communication about objectives, uncertainties and evidence levels is essential to maintain trust and manage expectations, particularly in self-pay or medical-tourism contexts where commercial pressures may be strong [31,104].

This structured, cyclic approach is illustrated schematically in Figure 4, which depicts the key decision points, intervention layers, and feedback mechanisms that characterize evidence-based functional longevity medicine.

## 8. Future Directions and Research Gaps

Despite compelling biological rationale and encouraging early data, the field of MSC-derived secretome and exosomes in LPRM remains in a formative stage, with several critical gaps that must be addressed to enable responsible clinical scaling. At the product level, there is an urgent need for standardized, consensus-driven criteria for characterizing secretome- and EV-based formulations, including minimal information requirements, robust potency assays and clear acceptance ranges for key components [29]. Existing efforts such as the MISEV2023 guidelines for extracellular vesicles provide an important starting point, but secretome products that combine vesicular and non-vesicular fractions pose additional challenges for analytical standardization and regulatory classification [29,105].

From a clinical-evidence perspective, well-designed randomized controlled trials are needed across the main longevity-relevant indications where preclinical and physician-led experiences suggest benefit—including chronic non-healing wounds, osteoarthritis and other degenerative joint diseases, neurodegenerative conditions and cardiometabolic syndromes [84]. These studies should incorporate appropriate comparators (including optimized standard of care), validated outcome measures that capture both symptom and function, and sufficiently long follow-up to assess durability of effect and late safety signals. Given the multi-system nature of aging, there is also a case for trials that evaluate composite outcomes such as frailty indices, global functional status or time to major adverse events, rather than focusing solely on disease-specific endpoints [106].

At the systems level, integrating secretome-based interventions into LPRM structures will require the development of frameworks that link biological-age metrics and risk stratification tools to rational selection and timing of regenerative treatment. Real-world data infrastructures, including multi-center registries and long-term follow-up programs, will be essential for capturing effectiveness and safety in heterogeneous populations, particularly in cross-border medical-tourism settings where continuity of care can be fragmented [31]. In parallel, regulators will need to refine classification schemes and approval pathways for secretome and exosomes, balancing the need for rigorous evaluation with proportional, innovation-friendly requirements.

Finally, ethical and communication challenges require explicit attention. As with many longevity-oriented interventions, there is a risk that secretome-based products could be over-promoted or positioned as universal rejuvenation remedies, particularly in commercial wellness environments. Transparent communication about evidence levels, realistic expectations and potential risks, along with efforts to promote equitable access as indications are established, will be critical to ensure that secretome-based LPRM evolves as a credible, patient-centered discipline rather than a niche for speculative therapies [104].

## 9. Longevity, Prevention and Medical Insurance

The growing recognition that healthspan—and not merely lifespan—is central to the sustainability of aging societies has significant implications for both private insurance companies and public healthcare systems [107,108]. Traditional insurance models have largely been constructed around reimbursing episodic, reactive care for manifest disease, with limited incentives to invest in long-term prevention or functional preservation. However, demographic aging, the rising prevalence of chronic multimorbidity and mounting fiscal pressures are pushing insurers toward more proactive, prevention-oriented approaches [109]. Concepts such as value-based insurance design (VBID) seek to align patient cost-sharing with the clinical value of services, reducing barriers to high-value preventive and chronic-care interventions while discouraging low-value utilization. In parallel, analyses of the longevity economy argue that enabling people to remain healthy, productive and independent for longer can generate substantial macroeconomic benefits and reduce strain on pension, long-term care and health-insurance systems [110]. Reports from industry and think-tanks highlight emerging models in which life, health and retirement products converge, offering integrated solutions that combine financial protection with access to prevention, digital health tools and healthy-aging programs [108].

Within this evolving landscape, LPRM-aligned interventions—including regenerative cell-free products—could eventually be incorporated into benefit designs for high-risk individuals, provided that robust evidence of cost-effectiveness and long-term safety is generated. For example, coverage for secretome-based wound-healing adjuncts in patients with diabetes and peripheral vascular disease might be justified if they reliably reduce amputation rates and hospitalizations; similarly, exosome-enriched interventions that demonstrably slow cognitive decline could be attractive in markets grappling with dementia-related costs [84]. In the nearer term, insurers may begin by supporting elements of LPRM that have clear evidence and lower regulatory barriers—such as biomarker-guided risk stratification, digital coaching and structured healthy-longevity programs—while monitoring developments in regenerative therapeutics [108,109].

In many markets, legacy product designs, short-term performance targets and regulatory constraints limit insurers’ willingness to make long-horizon preventive investments, even when modeling suggests long-term savings [108]. For regenerative interventions whose evidence is still emerging, this tension is especially acute. Over time, however, as data on the economic and clinical value of prevention-first strategies accumulate and as competitive pressures increase in aging societies, the alignment between insurers, health systems and LPRM providers is likely to strengthen [107]. Secretome-based regenerative strategies, if validated clinically and economically, may thus transition from a primarily self-pay niche toward inclusion in value-based, longevity-oriented mainstream benefit structures.

## 10. Conclusions

Regenerative medicine is undergoing a conceptual and practical shift from a narrow focus on cell transplantation toward a broader paradigm that emphasizes standardized, cell-free products derived from carefully characterized MSC sources. In parallel, longevity-oriented precision medicine has emerged as a framework for proactively managing aging trajectories through early risk detection, multi-modal interventions and personalization based on biomarkers, functional status and patient goals. The intersection of these two trends—captured in the construct of longevity-oriented precision regenerative medicine—offers a coherent pathway for integrating regenerative biology into preventive and healthspan-focused care.

Compared with existing reviews that focus either on MSC therapies in general or on extracellular vesicles in isolated indications, this article emphasizes the specific role of WJMSC-derived secretome and exosomes within LPRM and outlines an operational patient journey for healthspan extension.

Within this landscape, WJMSC products stand out as particularly compelling. They leverage an ethically sourced, highly proliferative neonatal cell population to generate a rich, multifactorial paracrine milieu that can be standardized, stored and delivered without the risks and challenges associated with live cell engraftment. Preclinical and early clinical evidence across osteoarthritis, systemic inflammation, chronic wounds and neurodegenerative conditions suggest that these acellular formulations can recapitulate many of the therapeutic benefits of MSC therapies while offering advantages in scalability, dosing control, safety and practical integration into outpatient, iterative treatment models.

Southeast Asia, and Thailand in particular, illustrates both the opportunities and the challenges of deploying such interventions in real-world settings. Evolving ATMP-aligned regulatory frameworks, advanced private-sector clinical infrastructure and a vibrant medical-tourism industry make the region an early adopter of LPRM-style programs that combine advanced diagnostics, lifestyle interventions and regenerative therapies. At the same time, these environments highlight the need for clear standards, robust evidence generation, transparent communication and ethical guardrails to prevent over-promising and to ensure that innovation translates into real, measurable benefit for patients.

Looking ahead, the maturation of MSC-derived secretome and exosome technologies will depend on continued advances in analytical characterization, manufacturing, clinical trial design and long-term safety monitoring, as well as alignment with evolving models of prevention-focused health systems and insurance. If these conditions are met, secretome-based regenerative strategies—anchored in MSC biology and integrated within structured LPRM pathways—have the potential to become a cornerstone of evidence-based longevity medicine, helping societies to shift the focus of healthcare from treating late-stage disease to maintaining function, independence and quality of life across the lifespan.

## Figures and Tables

**Table 1:** Comparative features of MSC-derived exosomes from different tissue sources [24,55]. Key features of exosomes derived from bone marrow, adipose tissue, WJ, and other perinatal MSC sources, highlighting why WJMSC-derived products are particularly attractive for scalable, longevity-oriented applications.

MSC Source	Advantages of Exosomes	Limitations of Exosomes	Example Indications or Contexts
**Bone marrow**	Well-characterized clinical track record; exosomes carry robust immunomodulatory and pro-angiogenic cargo; extensive preclinical data in cardiac, renal, and neurological models	Invasive harvest; donor age-related decline in MSC function and EV quality; more heterogeneity in donors with comorbidities	Cardiac and vascular repair, ischemic injury, graft-versus-host disease and immune modulation where adult donor material is acceptable
**Adipose** **derived**	Abundant tissue source; relatively easy collection by lipoaspiration; exosomes enriched for pro-angiogenic and matrix-modulating factors; attractive for musculoskeletal and dermal applications	Donor metabolic status (obesity, insulin resistance) may alter EV cargo; variability in collection and processing methods	Orthopedic/orthobiologic uses (osteoarthritis, tendon injuries), dermal and soft-tissue repair, aesthetic/dermatologic indications
**Wharton’s jelly**	Non-invasive collection from discarded perinatal tissue; young donor age; high proliferative capacity; exosomes and secretome often show stronger pro-regenerative and cytoprotective effects than adult MSC sources in wound-healing and skin models	Requires perinatal tissue banking/logistics; less long-term clinical follow-up than adult MSCs in some indications	LPRM programs, wound healing, skin and soft-tissue regeneration, allogeneic off-the-shelf products where scalability and low immunogenicity are priorities
**Other perinatal sources (cord blood/lining, placenta, amniotic fluid, etc.)**	Very low immunogenicity; rich developmental signaling profile; promising data for neuroprotection and organ protection in preclinical models	More complex tissue processing; heterogeneity between cell subsets; fewer standardized manufacturing platforms	Neuroprotection, neonatal/pediatric indications, organ protection in ischemia–reperfusion or inflammation-driven injury models

**Table 2:** Comparative attributes of live MSCs, exosome-enriched products and whole secretome products for longevity-oriented precision regenerative medicine (LPRM). Summary of key similarities and differences between live MSCs, exosome-enriched products and whole secretome across mechanistic, compositional, manufacturing, safety, practical and regulatory dimensions, highlighting trade-offs that are particularly relevant for iterative, outpatient LPRM applications [18,33,46].

Dimension	Live MSCs	Exosome-Enriched Fraction	Whole Secretome
**Primary mechanism of action**	Paracrine signaling (plus very limited engraftment and differentiation in some cases)	Paracrine signaling via small EV cargo (proteins, lipids, RNAs)	Broad paracrine signaling via soluble factors and extracellular vesicles
**Composition**	Viable, heterogeneous cell population (surface markers and secretome vary)	More defined subset of small EVs with selected cargo; highly reduced non-vesicular milieu	Complex mixture of proteins, lipids, nucleic acids, EVs and matrix fragments
**Dose definition**	Cell number (e.g., cells/kg); live cell number (with additional bioassays)	Particle count, protein load or EV-associated marker content per dose	Total protein/particle content; defined concentration per infusion/application
**Potency determination**	Technically challenging; high costs associated	Simple in vitro bioassay	Simple in vitro bioassay
**Manufacturing complexity**	High: cell sourcing, expansion, banking, viability and release testing	High: secretome production plus additional EV isolation and purification steps	Moderate: repeated harvests from stable MSC cultures; focus on secretome quality
**Scalability and productivity**	Limited by cell yield per donor and per passage	Moderate: inherits secretome scalability but extra processing can reduce yield	High: MSCs act as renewable “biofactories,” enabling multiple batches per bank
**Storage and logistics**	Cryopreserved cell products; strict cold chain and handling to preserve viability	Frozen EV preparations; stable but more sensitive to process and storage variables	Typically frozen or lyophilized; off-the-shelf reconstitution possible
**Dosing predictability**	Variable, influenced by in vivo viability, distribution and microenvironment	Higher: defined EV dose; functional output still depends on cargo consistency	Higher: defined ex vivo composition and dose; less dependent on in vivo cell fate
**Safety considerations**	Risks include microvascular obstruction, emboli, ectopic engraftment, immunogenicity and theoretical tumor support	Similarly to secretome; lower total protein load, but nanoscale biodistribution must be characterized	No viable cells; risks relate to bioactivity of cargo and residual impurities
**Regulatory classification (typical)**	Advanced therapy medicinal product/cell therapy product	Biologic/nanomedicine; emerging EV-specific regulatory guidance	Complex biologic; may be regulated as a biological drug or multi-API product
**Fit for iterative outpatient LPRM use**	Limited by long infusions, monitoring needs and cost per dose	Well suited but with higher cost of goods; best matched to high-value indications	Well suited: shorter infusions or local/topical use; amenable to maintenance regimens
**CNS and blood–brain barrier access**	Limited after intravenous delivery; invasive routes needed for direct CNS access	Strongest evidence for BBB interaction and CNS delivery among MSC-derived products	Can act indirectly via systemic immune-vascular modulation; some EV-mediated BBB interaction
**Example indications (current/near-term focus)**	Severe or refractory disease in controlled trials (e.g., GVHD, complex fistulae)	Neurodegenerative disease, stroke and TBI, targeted organ protection, diabetes	Chronic and acute wounds, osteoarthritis, early CNS and cardiometabolic prototypes

## Data Availability

No new data were created or analyzed in this study.

## Abbreviations

The following abbreviations are used in this manuscript:

MSC	Mesenchymal stem cell.
MSCs	Mesenchymal stem cells.
EVs	Extracellular vesicles.
UC	Umbilical cord.
WJ	Wharton’s jelly.
WJMSCs	Wharton’s jelly-derived mesenchymal stem cells.
GMP	Good Manufacturing Practice.
IL-6	Interleukin 6.
IL-8	Interleukin 8.
MCP-1	Monocyte chemoattractant protein 1.
VEGF	Vascular endothelial growth factor.
FGF	Fibroblast growth factor(s).
TGF-β	Transforming growth factor beta.
TIMPs	Tissue inhibitors of metalloproteinases.
CNS	Central nervous system.
BBB	Blood–brain barrier.
PRP	Platelet-rich plasma.
OA	Osteoarthritis.
T2DM	Type 2 diabetes mellitus.
STZ	Streptozotocin.
hUCMSC	Human umbilical cord mesenchymal stem cells.
IRS-1	Insulin receptor substrate 1.
HOMA-IR	Homeostasis Model Assessment—Insulin Resistance.
UC-MSCs	Umbilical cord mesenchymal stem cells.
ATMP	Advanced therapy medicinal product.
LPRM	Longevity-oriented precision regenerative medicine.
VBID	Value-based insurance design.
ADAS	Alzheimer’s Disease Assessment Scale.
MoCA	Montreal Cognitive Assessment.

## References

[1] Beard J.R., Officer A., de Carvalho I.A., Sadana R., Pot A.M., Michel J.-P., Lloyd-Sherlock P., Epping-Jordan J.E., Peeters G.M.E.E.G., Mahanani W.R. (2016). The World Report on Ageing and Health: A Policy Framework for Healthy Ageing. Lancet.

[2] World Health Organisation Noncommunicable Diseases. https://www.who.int/news-room/fact-sheets/detail/noncommunicable-diseases.

[3] Waldman S.A., Terzic A. (2019). Health Care Evolves from Reactive to Proactive. Clin. Pharmacol. Ther..

[4] Bischof E., Scheibye-Knudsen M., Siow R., Moskalev A. (2021). Longevity Medicine: Upskilling the Physicians of Tomorrow. Lancet Healthy Longev..

[5] Friebe M., Mittler-Matica R., Niestroj B. (2025). Investors’ Perspective on Healthspan in 2025. Qeios.

[6] Mironov S., Borysova O., Morgunov I., Zhou Z., Moskalev A. (2024). A Framework for an Effective Healthy Longevity Clinic. Aging Dis..

[7] Scott L.J. (2018). Darvadstrocel: A Review in Treatment-Refractory Complex Perianal Fistulas in Crohn’s Disease. BioDrugs.

[8] Fernández-Garza L.E., Barrera-Barrera S.A., Barrera-Saldaña H.A. (2023). Mesenchymal Stem Cell Therapies Approved by Regulatory Agencies around the World. Pharmaceuticals.

[9] US Federal Drug Association FDA Approves Remestemcel-L-Rknd for Steroid-Refractory Acute Graft Versus Host Disease in Pediatric Patients. https://www.fda.gov/drugs/resources-information-approved-drugs/fda-approves-remestemcel-l-rknd-steroid-refractory-acute-graft-versus-host-disease-pediatric.

[10] Hodgson-Garms M., Moore M.J., Martino M.M., Kelly K., Frith J.E. (2025). Proteomic Profiling of iPSC and Tissue-Derived MSC Secretomes Reveal a Global Signature of Inflammatory Licensing. Npj Regen. Med..

[11] Kehl D., Generali M., Mallone A., Heller M., Uldry A.-C., Cheng P., Gantenbein B., Hoerstrup S.P., Weber B. (2019). Proteomic Analysis of Human Mesenchymal Stromal Cell Secretomes: A Systematic Comparison of the Angiogenic Potential. Npj Regen. Med..

[12] Lalu M.M., McIntyre L., Pugliese C., Fergusson D., Winston B.W., Marshall J.C., Granton J., Stewart D.J. (2012). Canadian Critical Care Trials Group Safety of Cell Therapy with Mesenchymal Stromal Cells (SafeCell): A Systematic Review and Meta-Analysis of Clinical Trials. PLoS ONE.

[13] Baranovskii D.S., Klabukov I.D., Arguchinskaya N.V., Yakimova A.O., Kisel A.A., Yatsenko E.M., Ivanov S.A., Shegay P.V., Kaprin A.D. (2022). Adverse Events, Side Effects and Complications in Mesenchymal Stromal Cell-Based Therapies. Stem Cell Investig..

[14] Vulliet P.R., Greeley M., Halloran S.M., MacDonald K.A., Kittleson M.D. (2004). Intra-Coronary Arterial Injection of Mesenchymal Stromal Cells and Microinfarction in Dogs. Lancet.

[15] Volarevic V., Markovic B.S., Gazdic M., Volarevic A., Jovicic N., Arsenijevic N., Armstrong L., Djonov V., Lako M., Stojkovic M. (2018). Ethical and Safety Issues of Stem Cell-Based Therapy. Int. J. Med. Sci..

[16] Hwang W.Y.K., Bari S., Kawamata S., Choi B., Koaykul C., Huang H., Gupta P. (2025). Cell and Gene Therapy Approvals in Asia: Regulatory Landscape, Access and Affordability. Cytotherapy.

[17] Jeppesen D.K., Zhang Q., Franklin J.L., Coffey R.J. (2023). Extracellular Vesicles and Nanoparticles: Emerging Complexities. Trends Cell Biol..

[18] Vizoso F.J., Eiro N., Cid S., Schneider J., Perez-Fernandez R. (2017). Mesenchymal Stem Cell Secretome: Toward Cell-Free Therapeutic Strategies in Regenerative Medicine. Int. J. Mol. Sci..

[19] Meiliana A., Dewi N.M., Wijaya A. (2019). Mesenchymal Stem Cell Secretome: Cell-Free Therapeutic Strategy in Regenerative Medicine. Indones. Biomed. J..

[20] Kupcova Skalnikova H. (2013). Proteomic Techniques for Characterisation of Mesenchymal Stem Cell Secretome. Biochimie.

[21] Maguire G. (2013). Stem Cell Therapy without the Cells. Commun. Integr. Biol..

[22] Li X., Xiao H., Wang Z., Tang X., Yu X., Pan Y. (2024). Platelet Concentrates Preconditioning of Mesenchymal Stem Cells and Combined Therapies: Integrating Regenerative Strategies for Enhanced Clinical Applications. Cell Transplant..

[23] Trigo C.M., Rodrigues J.S., Camões S.P., Solá S., Miranda J.P. (2024). Mesenchymal Stem Cell Secretome for Regenerative Medicine: Where Do We Stand?. J. Adv. Res..

[24] Chin J.S., Tan M.L.L., Lim P.L.K., Sharma B., Yeo A., Aw Y.B., Ng Y.Z., Bonnard C., Becker D.L., Mok P. (2025). Secretome from Prolonged High-density Human Wharton’s Jelly Stem Cell Culture Accelerates Wound Healing in Both in Vitro and in Vivo Models. Int. Wound J..

[25] Fong C., Tam K., Cheyyatraivendran S., Gan S., Gauthaman K., Armugam A., Jeyaseelan K., Choolani M., Biswas A., Bongso A. (2014). Human Wharton’s Jelly Stem Cells and Its Conditioned Medium Enhance Healing of Excisional and Diabetic Wounds. J. Cell. Biochem..

[26] Bakhtyar N., Jeschke M.G., Mainville L., Herer E., Amini-Nik S. (2017). Acellular Gelatinous Material of Human Umbilical Cord Enhances Wound Healing: A Candidate Remedy for Deficient Wound Healing. Front. Physiol..

[27] Aw Y.B., Chen S., Yeo A., Dangerfield J.A., Mok P. (2024). Development and Functional Testing of a Novel in Vitro Delayed Scratch Closure Assay. Histochem. Cell Biol..

[28] Prado-Yupanqui J.W., Ramírez-Orrego L., Cortez D., Vera-Ponce V.J., Chenet S.M., Tejedo J.R., Tapia-Limonchi R. (2025). The Hidden Power of the Secretome: Therapeutic Potential on Wound Healing and Cell-Free Regenerative Medicine-A Systematic Review. Int. J. Mol. Sci..

[29] Théry C., Witwer K.W., Aikawa E., Alcaraz M.J., Anderson J.D., Andriantsitohaina R., Antoniou A., Arab T., Archer F., Atkin-Smith G.K. (2018). Minimal Information for Studies of Extracellular Vesicles 2018 (MISEV2018): A Position Statement of the International Society for Extracellular Vesicles and Update of the MISEV2014 Guidelines. J. Extracell. Vesicles.

[30] Witwer K.W., Soekmadji C., Hill A.F., Wauben M.H., Buzás E.I., Di Vizio D., Falcon-Perez J.M., Gardiner C., Hochberg F., Kurochkin I.V. (2017). Updating the MISEV Minimal Requirements for Extracellular Vesicle Studies: Building Bridges to Reproducibility. J. Extracell. Vesicles.

[31] Velasco R.P., Chaikledkaew U., Myint C.Y., Khampang R., Tantivess S., Teerawattananon Y. (2013). Advanced Health Biotechnologies in Thailand: Redefining Policy Directions. J. Transl. Med..

[32] Grand View Research Thailand Medical Tourism Market Size: Industry Report 2030. https://www.grandviewresearch.com/industry-analysis/thailand-medical-tourism-market-report.

[33] Hade M.D., Suire C.N., Suo Z. (2021). Mesenchymal Stem Cell-Derived Exosomes: Applications in Regenerative Medicine. Cells.

[34] Chouaib B., Haack-Sørensen M., Chaubron F., Cuisinier F., Collart-Dutilleul P.-Y. (2023). Towards the Standardization of Mesenchymal Stem Cell Secretome-Derived Product Manufacturing for Tissue Regeneration. Int. J. Mol. Sci..

[35] Collins H., Calvo S., Greenberg K., Forman Neall L., Morrison S. (2016). Information Needs in the Precision Medicine Era: How Genetics Home Reference Can Help. Interact. J. Med. Res..

[36] Shen X., Wang C., Zhou X., Zhou W., Hornburg D., Wu S., Snyder M.P. (2024). Nonlinear Dynamics of Multi-Omics Profiles during Human Aging. Nat. Aging.

[37] Bland J.S. (2022). Functional Medicine Past, Present, and Future. Integr. Med..

[38] Morgan A.A., Mooney S.D., Aronow B.J., Brenner S.E. (2016). Precision Medicine: Data and Discovery for Improved Health and Therapy. Pac. Symp. Biocomput..

[39] Park Y., Ha C., Lee C., Yoon Y.C., Park Y. (2017). Cartilage Regeneration in Osteoarthritic Patients by a Composite of Allogeneic Umbilical Cord Blood-Derived Mesenchymal Stem Cells and Hyaluronate Hydrogel: Results from a Clinical Trial for Safety and Proof-of-Concept with 7 Years of Extended Follow-Up. Stem Cells Transl. Med..

[40] Lee E.C., Ha T.W., Lee D.-H., Hong D.-Y., Park S.-W., Lee J.Y., Lee M.R., Oh J.S. (2022). Utility of Exosomes in Ischemic and Hemorrhagic Stroke Diagnosis and Treatment. Int. J. Mol. Sci..

[41] Bang O.Y., Kim E.H. (2019). Mesenchymal Stem Cell-Derived Extracellular Vesicle Therapy for Stroke: Challenges and Progress. Front. Neurol..

[42] Cortes-Galvez D., Dangerfield J.A., Metzner C. (2023). Extracellular Vesicles and Their Membranes: Exosomes vs. Virus-Related Particles. Membranes.

[43] Infante A., Rodríguez C.I. (2021). Cell and Cell-Free Therapies to Counteract Human Premature and Physiological Aging: MSCs Come to Light. J. Pers. Med..

[44] Fong C.Y., Richards M., Manasi N., Biswas A., Bongso A. (2007). Comparative Growth Behaviour and Characterization of Stem Cells from Human Wharton’s Jelly. Reprod. Biomed. Online.

[45] Witwer K.W., Théry C. (2019). Extracellular Vesicles or Exosomes? On Primacy, Precision, and Popularity Influencing a Choice of Nomenclature. J. Extracell. Vesicles.

[46] Harrell C.R., Fellabaum C., Jovicic N., Djonov V., Arsenijevic N., Volarevic V. (2019). Molecular Mechanisms Responsible for Therapeutic Potential of Mesenchymal Stem Cell-Derived Secretome. Cells.

[47] Chen P., Wang F., Ling B., Zhu Y., Lin H., Huang J., Wang X. (2025). Mesenchymal Stem Cell-Derived Extracellular Vesicles: Emerging Therapies for Neurodegenerative Diseases. Int. J. Nanomed..

[48] Keshtkar S., Azarpira N., Ghahremani M.H. (2018). Mesenchymal Stem Cell-Derived Extracellular Vesicles: Novel Frontiers in Regenerative Medicine. Stem Cell Res. Ther..

[49] Kou M., Huang L., Yang J., Chiang Z., Chen S., Liu J., Guo L., Zhang X., Zhou X., Xu X. (2022). Mesenchymal Stem Cell-Derived Extracellular Vesicles for Immunomodulation and Regeneration: A next Generation Therapeutic Tool?. Cell Death Dis..

[50] Birtwistle L., Chen X.-M., Pollock C. (2021). Mesenchymal Stem Cell-Derived Extracellular Vesicles to the Rescue of Renal Injury. Int. J. Mol. Sci..

[51] Ma Z.-J., Yang J.-J., Lu Y.-B., Liu Z.-Y., Wang X.-X. (2020). Mesenchymal Stem Cell-Derived Exosomes: Toward Cell-Free Therapeutic Strategies in Regenerative Medicine. World J. Stem Cells.

[52] Casati S., Giannasi C., Niada S., Della Morte E., Orioli M., Brini A.T. (2022). Lipidomics of Cell Secretome Combined with the Study of Selected Bioactive Lipids in an In Vitro Model of Osteoarthritis. Stem Cells Transl. Med..

[53] Goodarzi P., Alavi-Moghadam S., Payab M., Larijani B., Rahim F., Gilany K., Bana N., Tayanloo-Beik A., Foroughi Heravani N., Hadavandkhani M. (2019). Metabolomics Analysis of Mesenchymal Stem Cells. Int. J. Mol. Cell. Med..

[54] Song N., Scholtemeijer M., Shah K. (2020). Mesenchymal Stem Cell Immunomodulation: Mechanisms and Therapeutic Potential. Trends Pharmacol. Sci..

[55] Allouh M.Z., Rizvi S.F.A., Alamri A., Jimoh Y., Aouda S., Ouda Z.H., Hamad M.I.K., Perez-Cruet M., Chaudhry G.R. (2025). Mesenchymal Stromal/Stem Cells from Perinatal Sources: Biological Facts, Molecular Biomarkers, and Therapeutic Promises. Stem Cell Res. Ther..

[56] Chang C., Yan J., Yao Z., Zhang C., Li X., Mao H.-Q. (2021). Effects of Mesenchymal Stem Cell-Derived Paracrine Signals and Their Delivery Strategies. Adv. Healthc. Mater..

[57] Maacha S., Sidahmed H., Jacob S., Gentilcore G., Calzone R., Grivel J.-C., Cugno C. (2020). Paracrine Mechanisms of Mesenchymal Stromal Cells in Angiogenesis. Stem Cells Int..

[58] Burns A.B., Doris C., Vehar K., Saxena V., Bardliving C., Shamlou P.A., Phillips M.I. (2021). Novel Low Shear 3D Bioreactor for High Purity Mesenchymal Stem Cell Production. PLoS ONE.

[59] Sidhom K., Obi P.O., Saleem A. (2020). A Review of Exosomal Isolation Methods: Is Size Exclusion Chromatography the Best Option?. Int. J. Mol. Sci..

[60] Casorati B., Zafferri I., Castiglioni S., Maier J.A. (2025). Replicative Senescence in Mesenchymal Stem Cells: An In Vitro Study on Mitochondrial Dynamics and Metabolic Alterations. Antioxidants.

[61] Sears V., Ghosh G. (2020). Harnessing Mesenchymal Stem Cell Secretome: Effect of Extracellular Matrices on Proangiogenic Signaling. Biotechnol. Bioeng..

[62] Shabbir A., Cox A., Rodriguez-Menocal L., Salgado M., Badiavas E.V. (2015). Mesenchymal Stem Cell Exosomes Induce Proliferation and Migration of Normal and Chronic Wound Fibroblasts, and Enhance Angiogenesis In Vitro. Stem Cells Dev..

[63] Surowiecka A., Strużyna J. (2022). Adipose-Derived Stem Cells for Facial Rejuvenation. J. Pers. Med..

[64] Alam M., Hughart R., Champlain A., Geisler A., Paghdal K., Whiting D., Hammel J.A., Maisel A., Rapcan M.J., West D.P. (2018). Effect of Platelet-Rich Plasma Injection for Rejuvenation of Photoaged Facial Skin. JAMA Dermatol..

[65] Li C.-H., Zhao J., Zhang H.-Y., Wang B. (2023). Banking of Perinatal Mesenchymal Stem/Stromal Cells for Stem Cell-Based Personalized Medicine over Lifetime: Matters Arising. World J. Stem Cells.

[66] Butler M.G., Menitove J.E. (2011). Umbilical Cord Blood Banking: An Update. J. Assist. Reprod. Genet..

[67] Ardani Y., Indonesia University (2025). Mesenchymal Stromal/Stem Cells-Derived Exosomes (MSC-Exos) Therapy for Type 2 Diabetes Mellitus Patients. https://clinicaltrials.gov/study/NCT07144241.

[68] Xie X., Song Q., Dai C., Cui S., Tang R., Li S., Chang J., Li P., Wang J., Li J. (2023). Clinical Safety and Efficacy of Allogenic Human Adipose Mesenchymal Stromal Cells-Derived Exosomes in Patients with Mild to Moderate Alzheimer’s Disease: A Phase I/II Clinical Trial. Gen. Psychiatry.

[69] Haraszti R.A., Miller R., Stoppato M., Sere Y.Y., Coles A., Didiot M.-C., Wollacott R., Sapp E., Dubuke M.L., Li X. (2018). Exosomes Produced from 3D Cultures of MSCs by Tangential Flow Filtration Show Higher Yield and Improved Activity. Mol. Ther..

[70] Kapoor K.S., Harris K., Arian K.A., Ma L., Schueng Zancanela B., Church K.A., McAndrews K.M., Kalluri R. (2024). High Throughput and Rapid Isolation of Extracellular Vesicles and Exosomes with Purity Using Size Exclusion Liquid Chromatography. Bioact. Mater..

[71] Gámez-Valero A., Monguió-Tortajada M., Carreras-Planella L., Franquesa M., Beyer K., Borràs F.E. (2016). Size-Exclusion Chromatography-Based Isolation Minimally Alters Extracellular Vesicles’ Characteristics Compared to Precipitating Agents. Sci. Rep..

[72] Bari E., Perteghella S., Di Silvestre D., Sorlini M., Catenacci L., Sorrenti M., Marrubini G., Rossi R., Tripodo G., Mauri P. (2018). Pilot Production of Mesenchymal Stem/Stromal Freeze-Dried Secretome for Cell-Free Regenerative Nanomedicine: A Validated GMP-Compliant Process. Cells.

[73] Widowati W., Faried A., Gunanegara R.F., Rahardja F., Zahiroh F.H., Sutendi A.F., Nindya F.S., Azis R., Ekajaya R.K., Hadiprasetyo D.S. (2024). Wound Healing Potent of Lyophilized-Secretome Gel from Human Wharton’s Jelly Mesenchymal Stem Cells. Trends Sci..

[74] Sun Y., Liu G., Zhang K., Cao Q., Liu T., Li J. (2021). Mesenchymal Stem Cells-Derived Exosomes for Drug Delivery. Stem Cell Res. Ther..

[75] Aabling R.R., Alstrup T., Kjær E.M., Poulsen K.J., Pedersen J.O., Revenfeld A.L., Møller B.K., Eijken M. (2023). Reconstitution and Post-Thaw Storage of Cryopreserved Human Mesenchymal Stromal Cells: Pitfalls and Optimizations for Clinically Compatible Formulants. Regen. Ther..

[76] Nguyen T.T.-H., Phan K.T., Le P.T.-B., Pham P.V., Vu B.N. (2024). What Is the Optimal Timing and Solution for the Intravenous Infusion of Thawed Cryopreserved Mesenchymal Stem Cells?. Biomed. Res. Ther..

[77] Kearney C.M., Khatab S., van Buul G.M., Plomp S.G.M., Korthagen N.M., Labberté M.C., Goodrich L.R., Kisiday J.D., Van Weeren P.R., van Osch G.J.V.M. (2022). Treatment Effects of Intra-Articular Allogenic Mesenchymal Stem Cell Secretome in an Equine Model of Joint Inflammation. Front. Vet. Sci..

[78] Mocchi M., Bari E., Marrubini G., Bonda A.F., Perteghella S., Tartara F., Cofano F., Perna G.d., Giovannelli L., Mandracchia D. (2021). Freeze-Dried Mesenchymal Stem Cell-Secretome Pharmaceuticalization: Optimization of Formulation and Manufacturing Process Robustness. Pharmaceutics.

[79] Terstappen G.C., Meyer A.H., Bell R.D., Zhang W. (2021). Strategies for Delivering Therapeutics across the Blood-Brain Barrier. Nat. Rev. Drug Discov..

[80] Achar A., Myers R., Ghosh C. (2021). Drug Delivery Challenges in Brain Disorders across the Blood–Brain Barrier: Novel Methods and Future Considerations for Improved Therapy. Biomedicines.

[81] Gugliandolo A., Bramanti P., Mazzon E. (2020). Mesenchymal Stem Cells in Multiple Sclerosis: Recent Evidence from Pre-Clinical to Clinical Studies. Int. J. Mol. Sci..

[82] Jo C.H., Lee Y.G., Shin W.H., Kim H., Chai J.W., Jeong E.C., Kim J.E., Shim H., Shin J.S., Shin I.S. (2014). Intra-Articular Injection of Mesenchymal Stem Cells for the Treatment of Osteoarthritis of the Knee: A Proof-of-Concept Clinical Trial. Stem Cells.

[83] Sadyah N.A.C., Heri-Nugroho N., Putra A., Riwanto I. (2025). Secretome of Human MSC Gel Improves DFU Healing through NF-kB P50 and CD163 mRNA Expression. Pak. J. Biol. Sci. PJBS.

[84] Suhandi C., Wilar G., Elamin K.M., Dewayani A.R., Ghaliya S., Abdullah A., Wathoni N. (2025). The Effect of Stem Cell Secretome on the Improvement of Diabetic Wound Recovery: A Systematic Review and Meta-Analysis of In Vivo Studies. Curr. Ther. Res. Clin. Exp..

[85] Li L., Chen X., Wang W.E., Zeng C. (2016). How to Improve the Survival of Transplanted Mesenchymal Stem Cell in Ischemic Heart?. Stem Cells Int..

[86] Sylakowski K., Bradshaw A., Wells A. (2020). Mesenchymal Stem Cell/Multipotent Stromal Cell Augmentation of Wound Healing. Am. J. Pathol..

[87] Prasai A., Jay J.W., Jupiter D., Wolf S.E., El Ayadi A. (2022). Role of Exosomes in Dermal Wound Healing: A Systematic Review. J. Investig. Dermatol..

[88] Qin X., He J., Wang X., Wang J., Yang R., Chen X. (2023). The Functions and Clinical Application Potential of Exosomes Derived from Mesenchymal Stem Cells on Wound Repair: A Review of Recent Research Advances. Front. Immunol..

[89] Zhao H., Li Z., Wang Y., Zhou K., Li H., Bi S., Wang Y., Wu W., Huang Y., Peng B. (2023). Bioengineered MSC-Derived Exosomes in Skin Wound Repair and Regeneration. Front. Cell Dev. Biol..

[90] ElSayed N.A., Aleppo G., Aroda V.R., Bannuru R.R., Brown F.M., Bruemmer D., Collins B.S., Hilliard M.E., Isaacs D., Johnson E.L. (2023). Classification and Diagnosis of Diabetes: Standards of Care in Diabetes-2023. Diabetes Care.

[91] Skyler J.S., Fonseca V.A., Segal K.R., Rosenstock J., MSB-DM003 Investigators (2015). Allogeneic Mesenchymal Precursor Cells in Type 2 Diabetes: A Randomized, Placebo-Controlled, Dose-Escalation Safety and Tolerability Pilot Study. Diabetes Care.

[92] Sun Y., Shi H., Yin S., Ji C., Zhang X., Zhang B., Wu P., Shi Y., Mao F., Yan Y. (2018). Human Mesenchymal Stem Cell Derived Exosomes Alleviate Type 2 Diabetes Mellitus by Reversing Peripheral Insulin Resistance and Relieving β-Cell Destruction. ACS Nano.

[93] Jiao Y.-R., Chen K.-X., Tang X., Tang Y.-L., Yang H.-L., Yin Y.-L., Li C.-J. (2024). Exosomes Derived from Mesenchymal Stem Cells in Diabetes and Diabetic Complications. Cell Death Dis..

[94] Mahdipour E., Salmasi Z., Sabeti N. (2019). Potential of Stem Cell-Derived Exosomes to Regenerate β Islets through Pdx-1 Dependent Mechanism in a Rat Model of Type 1 Diabetes. J. Cell. Physiol..

[95] Li L., Li J., Guan H., Oishi H., Takahashi S., Zhang C. (2023). Human Umbilical Cord Mesenchymal Stem Cells in Diabetes Mellitus and Its Complications: Applications and Research Advances. Int. J. Med. Sci..

[96] Li F.-X.-Z., Lin X., Xu F., Shan S.-K., Guo B., Lei L.-M., Zheng M.-H., Wang Y., Xu Q.-S., Yuan L.-Q. (2021). The Role of Mesenchymal Stromal Cells-Derived Small Extracellular Vesicles in Diabetes and Its Chronic Complications. Front. Endocrinol..

[97] Zang L., Hao H., Liu J., Li Y., Han W., Mu Y. (2017). Mesenchymal Stem Cell Therapy in Type 2 Diabetes Mellitus. Diabetol. Metab. Syndr..

[98] Wang Y., Chen H., Li Y., Hao H., Liu J., Chen Y., Meng J., Zhang S., Gu W., Lyu Z. (2024). Predictive Factors That Influence the Clinical Efficacy of Umbilical Cord-Derived Mesenchymal Stromal Cells in the Treatment of Type 2 Diabetes Mellitus. Cytotherapy.

[99] Widyaningsih W., Putra A., Priyantini S., Muhar A.M., Sumarawati T., Trisnadi S., Amalina N.D., Alif I., Prasetio A., Irawan R.C.S. (2024). Secretome of Hypoxia-Preconditioned Mesenchymal Stem Cells Ameliorates Hyperglycemia in Type 2 Diabetes Mellitus Rats. Trends Sci..

[100] (2021). Ruijin Hospital Open-Label, Single-Center, Phase I/II Clinical Trial to Evaluate the Safety and the Efficacy of Exosomes Derived from Allogenic Adipose Mesenchymal Stem Cells in Patients with Mild to Moderate Dementia Due to Alzheimer’s Disease; clinicaltrials.gov. https://clinicaltrials.gov/study/NCT04388982.

[101] Phelps J., Orr A., Elvira K.S., Willerth S.M. (2025). Extracellular Vesicles for the Treatment of Alzheimer’s Disease: A Systematic Review. J. Extracell. Biol..

[102] Santamaria G., Brandi E., Vitola P.L., Grandi F., Ferrara G., Pischiutta F., Vegliante G., Zanier E.R., Re F., Uccelli A. (2021). Intranasal Delivery of Mesenchymal Stem Cell Secretome Repairs the Brain of Alzheimer’s Mice. Cell Death Differ..

[103] Hua Z., Zhou N., Zhou Z., Fu Z., Guo R., Akogo H.Y., Yang J., Yu M., Jiang Y., Lan S. (2025). Intranasal Administration of Stem Cell Derivatives for the Treatment of AD Animal Models: A Systematic Review and Meta-Analysis. Stem Cell Res. Ther..

[104] Gopalan N., Mohamed Noor S.N., Salim Mohamed M. (2021). The Pro-Medical Tourism Stance of Malaysia and How It Affects Stem Cell Tourism Industry. Sage Open.

[105] Welsh J.A., Goberdhan D.C.I., O’Driscoll L., Buzas E.I., Blenkiron C., Bussolati B., Cai H., Di Vizio D., Driedonks T.A.P., Erdbrügger U. (2024). Minimal Information for Studies of Extracellular Vesicles (MISEV2023): From Basic to Advanced Approaches. J. Extracell. Vesicles.

[106] Moqri M., Herzog C., Poganik J.R., Justice J., Belsky D., Higgins-Chen A., Moskalev A., Fuellen G., Cohen A.A., Bautmans I. (2023). Biomarkers of Aging for the Identification and Evaluation of Longevity Interventions. Cell.

[107] World Economic Forum Longevity Economy Principles: The Foundation for a Financially Resilient Future. https://www3.weforum.org/docs/WEF_Longevity_Economy_Principles_2024.pdf.

[108] Capgemini Research Institute World Life Insurance Report 2023: The Aging Well Opportunity. https://www.capgemini.com/wp-content/uploads/2023/10/WLIR_2023_web.pdf.

[109] UN Environment Programme Finance Initiative Principles for Sustainable Insurance: Health Is Our Greatest Wealth. https://www.unepfi.org/wordpress/wp-content/uploads/2023/06/Health-is-Our-Greatest-Wealth_-How-life-health-insurers-can-drive-better-health-outcomes-and-address-the-protection-gap.pdf.

[110] Fendrick M.A. (2024). Value-Based Insurance Design: Aligning Patient and Provider Incentives to Increase Use of Highvalue Care, Enhance Equity, and Eliminate Low Value Services. https://vbidcenter.org/wp-content/uploads/2024/01/VBID2024.pdf.

